# Urease-powered nanobots for radionuclide bladder cancer therapy

**DOI:** 10.1038/s41565-023-01577-y

**Published:** 2024-01-15

**Authors:** Cristina Simó, Meritxell Serra-Casablancas, Ana C. Hortelao, Valerio Di Carlo, Sandra Guallar-Garrido, Sandra Plaza-García, Rosa Maria Rabanal, Pedro Ramos-Cabrer, Balbino Yagüe, Laura Aguado, Lídia Bardia, Sébastien Tosi, Vanessa Gómez-Vallejo, Abraham Martín, Tania Patiño, Esther Julián, Julien Colombelli, Jordi Llop, Samuel Sánchez

**Affiliations:** 1 Center for Cooperative Research in Biomaterials (CIC biomaGUNE), Basque Research and Technology Alliance (BRTA), Donostia–San Sebastián, Spain; 2 Institute for Bioengineering of Catalonia (IBEC), The Barcelona Institute for Science and Technology (BIST), Barcelona, Spain; 3 Departament de Genètica i de Microbiologia, Facultat de Biociències, Universitat Autònoma de Barcelona, Barcelona, Spain; 4 Unitat de Patologia Murina i Comparada, Department of Animal Medicine and Surgery, Veterinary Faculty, Universitat Autònoma de Barcelona, Barcelona, Spain; 5 IKERBASQUE, Basque Foundation for Science, Bilbao, Spain; 6 Laboratory of Neuroimaging and Biomarkers of Inflammation, Achucarro Basque Center for Neuroscience, Leioa, Spain; 7 Institute for Research in Biomedicine (IRB Barcelona), The Barcelona Institute of Science and Technology (BIST), Barcelona, Spain; 8 Biomedical Engineering Department, Institute for Complex Molecular Systems, Technische Universiteit Eindhoven, Eindhoven, The Netherlands; 9 Institució Catalana de Recerca i Estudis Avançats (ICREA), Barcelona, Spain; 10 Present Address: Department of Radiology, Mallinckrodt Institute of Radiology, Washington University School of Medicine in St. Louis, St Louis, MO USA; 11 Present Address: Department of Biochemistry, University of Geneva, Geneva, Switzerland; 12 Present Address: Department of Biomedical Sciences, Faculty Of Health Sciences, University of Copenhagen, Copenhagen, Denmark

**Keywords:** Nanotechnology in cancer, Imaging techniques and agents, Nanoparticles

## Abstract

Bladder cancer treatment via intravesical drug administration achieves reasonable survival rates but suffers from low therapeutic efficacy. To address the latter, self-propelled nanoparticles or nanobots have been proposed, taking advantage of their enhanced diffusion and mixing capabilities in urine when compared with conventional drugs or passive nanoparticles. However, the translational capabilities of nanobots in treating bladder cancer are underexplored. Here, we tested radiolabelled mesoporous silica-based urease-powered nanobots in an orthotopic mouse model of bladder cancer. In vivo and ex vivo results demonstrated enhanced nanobot accumulation at the tumour site, with an eightfold increase revealed by positron emission tomography in vivo. Label-free optical contrast based on polarization-dependent scattered light-sheet microscopy of cleared bladders confirmed tumour penetration by nanobots ex vivo. Treating tumour-bearing mice with intravesically administered radio-iodinated nanobots for radionuclide therapy resulted in a tumour size reduction of about 90%, positioning nanobots as efficient delivery nanosystems for bladder cancer therapy.

## Abstract (web summary)

Bladder cancer treatment suffers from low therapeutic efficacy. Here the authors present radioactive ^131^I-labelled urease-powered nanobots that exhibit enhanced accumulation at the tumour site, enabling effective radionuclide therapy at low doses as an alternative treatment option for bladder cancer.

## Main

Bladder cancer remains a major health concern worldwide. At diagnosis, approximately 75% of cases are non-muscle-invasive bladder cancer^1^, which is treated by intravesical administration of immunotherapeutic (*Mycobacterium bovis* Bacillus Calmette–Guérin (BCG)^2^) and/or chemotherapeutic agents (mitomycin C^3^), after transurethral resection of the tumour. These treatments cause adverse side effects and have limited effectiveness, as evidenced by 5 yr recurrence rates of 30–70% (ref. ^4^) and progression rates of 10–30% (ref. ^5^). Therefore, bladder cancer patients require frequent cystoscopic surveillance and retreatment, making bladder cancer one of the most expensive malignancies^5^.

The efficacy of classical treatment approaches is limited by several factors, including the sedimentation of therapeutic agents and the continuous addition of fresh urine, which prevents them from diffusing evenly through the whole bladder volume^6^, poor retention in the bladder and weak adhesion to the target site^7^. In contrast to the standard non-muscle-invasive bladder cancer treatment, the non-motile BCG, ideal treatments should employ simple biocompatible systems capable of reaching and penetrating the tumour mass without leaving subregions untreated, which may lead to recurrence. In this context, self-propelled nanoparticles (hereafter called nanobots)^8–11^ have emerged as delivery systems of therapeutic agents. These motion capabilities allow them to navigate complex fluids and overcome biological matrices^12–18^, typically a major obstacle for drugs and passive nanoparticles. Chemically powered nanobots convert chemical energy from the surrounding fluid into mechanical propulsion, with enzyme nanobots able to take up endogenous substrates, opening possibilities for biomedical applications^19–21^. We have previously shown that urease-powered nanobots based on mesoporous silica nanoparticles (MSNPs) display an enhanced motion, with propulsion resulting from an ionic gradient of ammonia and CO_2_ created by the asymmetric decomposition of urea around the particle^22–24^.

The field of medical micro- and nanomotors is rapidly advancing towards in vivo applications^18,25–27^, with their collective motion successfully tracked in mouse bladders using various imaging techniques such as fluorescence^28^, photoacoustic^29,30^ and magnetic resonance imaging (MRI)^31,32^ or positron emission tomography–computed tomography (PET-CT)^33^. This collective swarming behaviour promotes convection and mixing inside the bladder, thus retarding nanobot sedimentation while ensuring a homogeneous distribution even with the entrance of fresh urine^33^. However, the translation of nanobots into practical applications for treating bladder cancer and other types of cancer is still in its early stages. Despite recent advancements in nanobot development and initial proof-of-concept studies in biomedicine, their practical use for in vivo tumour therapy remains limited^31,34–37^. Nevertheless, the ability of nanobots to navigate holds the potential for them to serve as carriers of active therapeutic agents, such as alpha or beta emitters for radionuclide therapy (RNT)^38^. RNT relies on the targeted delivery of cytotoxic radiation from within the body. The recent approval of Lutathera^39^ and Pluvicto^40^ for the treatment of neuroendocrine tumours and metastatic castration-resistant prostate cancer, respectively, have boosted clinical interest in RNT. For bladder cancer, the limited efficacy of standard therapeutic approaches and the possibility of utilizing carriers to maximize accumulation, penetration and retention within the tumour, thereby minimizing off-target accumulation and side effects, highlights the potential of RNT as a promising therapeutic alternative.

Here, we use an orthotopic mouse model of bladder cancer to comprehensively characterize the successful accumulation of urease-powered nanobots using in vivo and ex vivo imaging techniques after intravesical injection to mimic clinical practice. Moreover, the enhanced accumulation enables ^131^I-labelled urease-powered nanobots to have a therapeutic effect at doses substantially below those required for passive particles to be efficient. These results take us one step closer toward translational applications and highlight the underexplored potential of nanobots for innovative bladder cancer treatment options.

## Nanobot fabrication and characterization

**Fig. 1:**
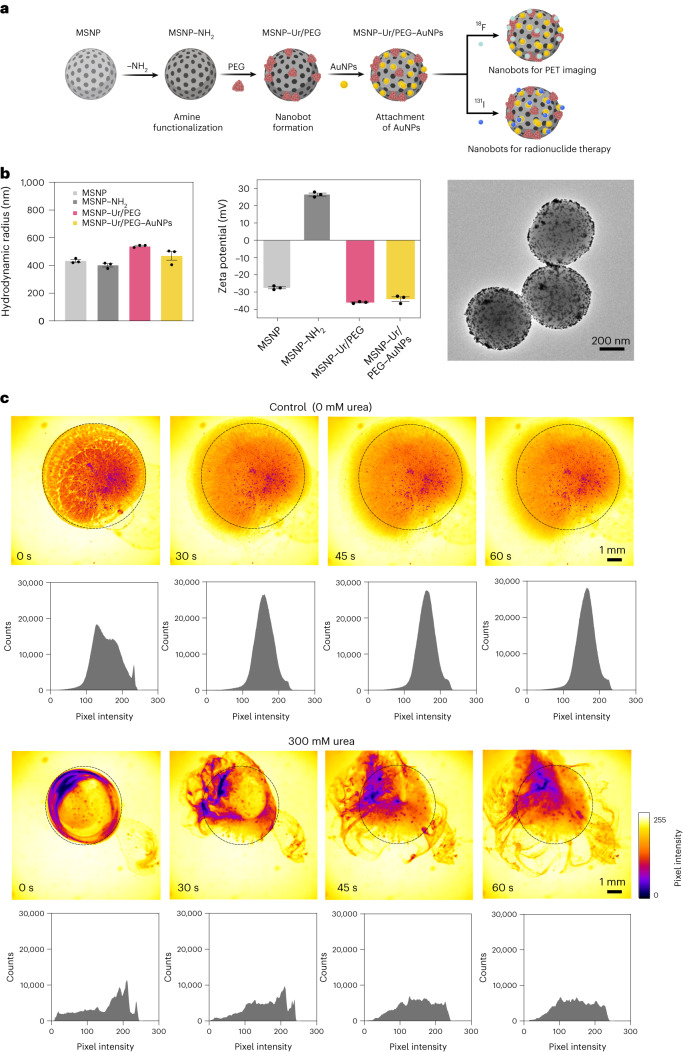
Fabrication, radiolabelling, characterization and motion dynamics of urease-powered nanobots. **a**, Schematic representation of the nanobot fabrication process and radiolabelling. Ur, urease. **b**, Left: nanobot characterization by dynamic light scattering (*n* = 3, technical replicates). Data are presented as mean values and error bars represent the s.e.m. Centre: zeta potential (*n* = 3, technical replicates) Data are presented as mean values and error bars represent the s.e.m. Right: transmission electron microscopy image. **c**, Snapshots depicting the nanobot motion dynamics in the absence and presence (300 mM) of urea as fuel, and corresponding pixel intensity histograms for the ROI marked by a circle. Panel **a** created with BioRender.com. Source data

Urease-powered nanobots based on approximately 450-nm-sized MSNPs were prepared using a modified Stöber method^41^ (Methods) and following a previously reported process for enzyme functionalization^33^. Briefly, the MSNP surfaces were modified with amine groups by attaching aminopropyltriethoxysilane (APTES) and then activated with glutaraldehyde. The latter acted as a crosslinker between the MSNPs and urease (or BSA in the control case) and heterobifunctional polyethylene glycol (PEG) molecules (Fig. 1a). The resulting nanobots were decorated with gold nanoparticles (AuNPs) on their surfaces. To allow quantification by PET imaging, nanobots and BSA–nanoparticles (BSA-NPs) were labelled with the positron emitter ^18^F (Fig. 1a). Radiofluorination was achieved following an established procedure^33^ based on the condensation of a prelabelled prosthetic group (6-[^18^F]fluoronicotinic acid 2,3,5,6-tetrafluorophenyl ester, [^18^F]F-PyTFP) with the amino groups present on the enzyme. Comparing the enzymatic activity of radiolabelled and non-radiolabelled nanobots, we found that ^18^F did not decrease activity (Supplementary Fig. 1).

We monitored the hydrodynamic radius and surface charge of the nanoparticles at each fabrication step using dynamic light scattering, revealing a slight size change in each functionalization step attributed to the addition of molecules on their surfaces (Fig. 1b, left panel). Zeta potentials followed the expected behaviour, reaching a final value of −34.5 ± 1.3 mV, which can be explained by urease’s isoelectric point of 6 and the net charge of AuNPs^42^ (Fig. 1b, middle panel). Transmission electron microscopy further confirmed the distribution of AuNPs on the surface of the nanobots (Fig. 1b, right panel).

To study the in vitro dynamics and collective behaviour of nanobots, we added a droplet of nanobots to a PBS solution with and without 300 mM urea. In the presence of urea, nanobots formed active and vigorous fronts and three-dimensional (3D) vortices (Supplementary Video 1). In contrast, without urea, they sedimented near the addition site, exhibiting a typical two-dimensional (2D) dispersion pattern on the glass substrate (Supplementary Video 2), as previously reported^33^. We quantified their collective behaviour by defining a region of interest (ROI) corresponding to the area occupied by the nanobots at time zero and plotting the pixel intensity at each time point (Fig. 1c). Without urea, the histograms are typical for passive diffusion, with a narrow central peak and almost no change in 1 min. With urea, the distribution quickly becomes more heterogeneous (illustrated by a broadening pixel intensity spectrum), with nanobots accumulating in certain regions (purple or dark-red colours in Fig. 1c). This uneven distribution and 3D dynamics aligns with the expected behaviour associated with swarming and will be beneficial to overcome limitations of existing bladder cancer treatments such as sedimentation and low volume dispersion, as seen in the control experiment without urea. To demonstrate the impact of swarm motion on mass transport, we tracked 2-µm-sized tracer particles initially dispersed evenly in PBS or 300 mM urea. After introducing nanobots, we monitored swarm formation while tracking tracer particles. In PBS, swarms showed slight expansion via passive diffusion, leading to minor linear deviations in tracer particle trajectories. In contrast, nanobots in urea (Supplementary Fig. 2 and Supplementary Video 4) significantly increased tracer particle displacement, as seen in trajectory patterns and mean square displacement analysis. Tracer particles exhibited coordinated movement with the swarm, resulting in complex 3D trajectories.

## Orthotopic murine model of bladder cancer

**Fig. 2:**
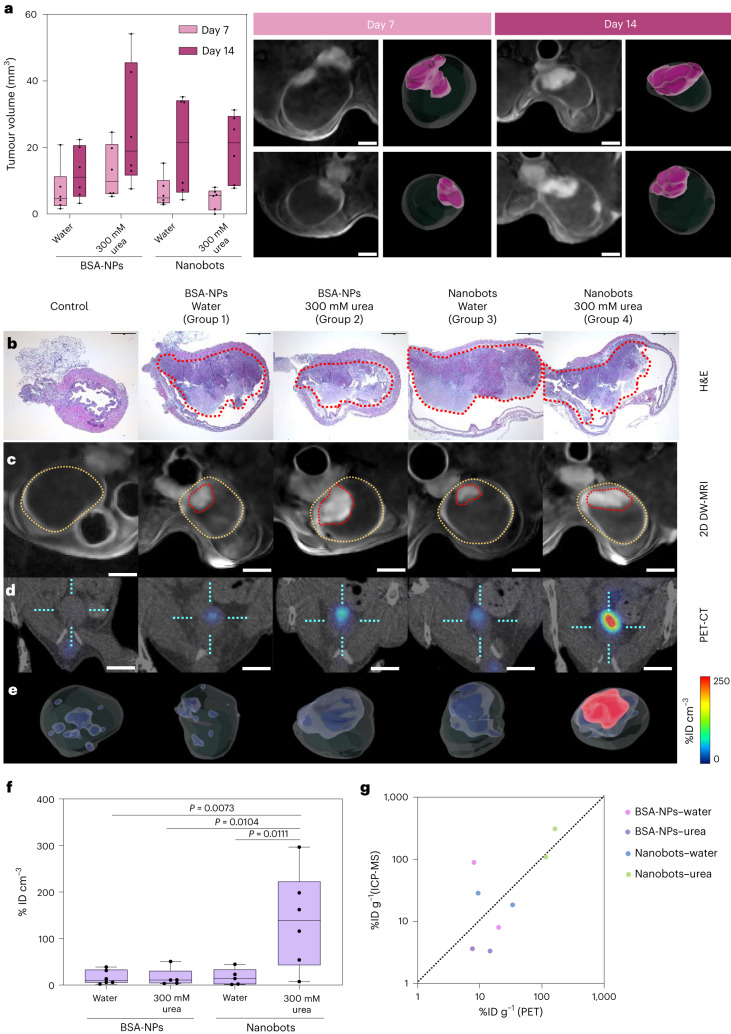
In vivo studies of nanobot accumulation in a bladder cancer orthotopic murine model. **a**, Left: tumour volumes (determined by MRI) on days 7 and 14 after cell implantation for the different study groups (*n* = 6 per group, biological replicates). Results are expressed in a box plot (centre line at the median; upper and lower bounds at 75th and 25th percentiles, respectively; one dot per animal) with whiskers at minimum and maximum values. Right: 2D DW-MRI images of the bladder (hypointense circular region) of two representative mice at *t* = 7 and 14 days after inoculation of MB49 cells. Scale bars, 2 mm. 3D renders of whole bladders (transparent) and tumours (purple) are presented next to each MRI image. **b**, Haematoxylin–eosin stains showing bladders containing tumours (delineated by red dotted lines) for one representative animal per group. Scale bars, 1 mm. **c**, 2D DW-MRI images of the bladder (hypointense circular region) of one representative animal per study group. Tumours appear hyperintense (tumours delineated by a red dotted line and bladders in yellow). Scale bars 2 mm. **d**, Coronal PET 2D images overlaid on CT images of one representative animal per study group. The dotted cyan cross shows the bladder position and the radioactive intensity has been colour-coded (given as the percentage of injected dose per millilitre, %ID cm^−^^3^). Scale bars, 5 mm. **e**, 3D renders of the whole bladder (transparent) and radioactive signal accumulation (colour-coded) of the PET-CT images shown in **d**. **f**, Box (centre line at the median; upper and lower bounds at 75th and 25th percentiles, respectively; one dot per animal) and whisker (minimum and maximum values) plot of radioactivity accumulation normalized by tumour volume (determined by MRI) in animals from all groups (*n* = 6 for BSA-NPs in water and nanobots in urea, *n* = 5 for the other groups, biological replicates), shown as percentages of the injected dose per cubic centimetre of tumour (%ID cm^−3^). Statistical analysis was performed via one-way analysis of variance (ANOVA). **g**, Correlation of tumour accumulation obtained by PET and ICP-MS results for all groups (*n* = 2 per group, biological replicates). Source data

We used an orthotopic syngeneic murine model to evaluate the abiliity of nanobots to reach the tumour site (Methods). This model has been widely used in therapeutic experiments^43^, allowing the tumour to grow in an anatomically representative tissue microenvironment. Tumour volume was monitored by MRI on days 7 and 14 after cell implantation (Fig. 2a and Supplementary Fig. 3). Diffusion-weighted magnetic resonance imaging (DW-MRI) images clearly showed the tumour as a grey mass in the upper part of the bladder (Fig. 2a). Mice were randomized among the four groups according to tumour volume (Table 1). The control group consisted of non-tumour-bearing mice with ^18^F-nanobots administered in 300 mM urea as the vehicle.

**Table 1:** Summary of the different experimental scenarios performed in vivo

Group	*n*^a^	Compound	Vehicle
Control	3	^18^F-nanobots	Urea
1	6	^18^F-BSA-NPs	Water
2	5	^18^F-BSA-NPs	Urea
3	5	^18^F-nanobots	Water
4	6	^18^F-nanobots	Urea

^a^Six animals were inoculated per group except for control (*n* = 3); the ones listed in the table are those that showed tumour growth and were included in the study.

The histopathological assessment of the tumours using haematoxylin–eosin staining after PET imaging confirmed the presence of a solid mass (corresponding to tumour) in tumour-bearing mice, under the lamina propria and growing to the lumen of the bladder (Fig. 2b). Lamina propria infiltration by mononuclear and polymorphonuclear inflammatory cells was observed, as is usual in this model. No differences were observed among groups (Fig. 2b, groups 1–4, and Supplementary Fig. 4). 2D DW-MRI acquired through the bladder indicated that tumours were hyperintense (Fig. 2c).

We explored the accumulation of nanobots in bladder tumour using PET imaging with CT for anatomical reference (Fig. 2d). For PET analysis, labelled nanobots and vehicle solutions (water or urea) were intravesically administered for 1 h. PET-CT acquisitions were performed 3 h after administration and immediately after bladder evacuation to ensure that the radioactive signal exclusively originated from the nanobots attached to the tumour. Administering ^18^F-nanobots in urea in non-tumour-bearing mice resulted in a negligible fraction (about 0.02% of the injected dose) of radioactivity being observed, indicating the low adherence and/or penetration capacity of the labelled nanobots in a healthy bladder (Fig. 2d,e).

In tumour-bearing animals, PET images showed that radioactive signals were collocated with tumour positions as determined via MRI (Fig. 2d; groups 1–4). Radioactivity was primarily at the target site, with lower percentages in some animals visible in the stomach, liver and kidneys, probably due to ingestion of radioactivity during the awake period (between dose administration and image acquisition) (Supplementary Fig. 5). Importantly, only animals injected with nanobots and 300 mM of urea showed substantial accumulation of radiotracer in the tumour mass (Fig. 2d,e). With urea, we observed higher accumulations of ^18^F-nanobots (group 4; about 2.5%ID—percentage of injected dose) when compared with the control and groups 1, 2 and 3. In contrast, ^18^F-nanobots administered in water (group 3) and ^18^F-BSA-NPs administered in water and urea (groups 1 and 2, respectively) showed average tumour uptake below 1%ID (Supplementary Fig. 5). Normalizing radioactivity accumulation to tumour volume confirmed significantly higher tumour uptake of ^18^F-nanobots administered in urea (group 4; about 150%ID cm^−^^3^) when compared with groups 1, 2 and 3 (Fig. 2f), thus confirming the benefits of active motion for tumour accumulation. In fact, values for groups 1–3 were statistically equivalent, suggesting that effective motion requires a urease–urea combination while other non-catalytically active combinations do not promote tumour uptake.

Stability studies performed in urine extracted from tumour-bearing animals demonstrated that radiochemical purity of the labelled nanobots was close to 60% after 1 h of incubation, suggesting a certain label detachment. Notably, stability in ultrapure water and urea solution showed radiochemical purity of >90% after 1 h of incubation. Considering this, we performed ex vivo inductively coupled plasma mass spectrometry (ICP-MS) analyses to determine the concentration of gold and hence confirm that the PET signal was due to the particles and not from the detached radioisotope. The correlation between PET and ICP-MS confirmed that partial radiolabel detachment from nanobots in pure urine (in vivo) did not affect the determination of tumour accumulation (Fig. 2g). However, the slight discrepancies observed between the two techniques could be due to inaccuracies in delineating the volumes of interest in the PET images or partial volume effects, as bladder tumours are relatively small when compared with the spatial resolution of PET systems.

## Scattered light-sheet microscopy locates nanobots ex vivo

We employed optical microscopy techniques to pinpoint the location of nanobots in bladders and assess tumour penetration with a spatial precision that exceeds the known limits of PET. Unfortunately, confocal microscopy imaging of thin histological sections (Supplementary Information and Supplementary Fig. 6) could not be used for nanobot detection, as the high levels of bladder tissue and tumour autofluorescence would drown out both intrinsic fluorescence and secondary fluorescence labelling. Hence, we hypothesized that nanobots could be detectable by elastic scattering, and we aimed to image AuNP-decorated nanobots using scattered light-sheet microscopy (sLS), an emerging, label-free imaging approach based on light-sheet technology. We used a custom light-sheet microscope^44,45^ for whole-organ imaging (Supplementary Fig. 7) that, combined with tissue clearing, can overcome histological section limitations and analyse nanobot tumour penetration in three dimensions. Using conventional light-sheet imaging to detect fluorescein isothiocyanate (FITC)-fluorescent nanobots was again challenging. First, their fluorescence is highly unstable in an optically cleared environment (Supplementary Fig. 8f,g). Second, it is too weak, compared with the high bladder tissue autofluorescence, and the FITC signal did not yield sufficient signal to noise ratio in tissues. To address these issues and avoid challenges related to secondary label penetration in whole-mount tissues, we implemented a tailored version of sLS for the detection of nanobots against the background noise of reflected laser light. In brief, we identified the best combination of light polarization for illumination and detection (Supplementary Information and Supplementary Fig. 8), obtaining detectable signals of individual 450 nm nanobots in vitro even at moderate (9.6×) magnification. Next, we characterized cleared bladder tissues using sLS, with and without tumour, and with and without nanobots, with results demonstrating that sLS can detect nanobots in whole cleared tissues (Supplementary Figs. 8 and 9), and that tight polarization control is essential for distinguishing between nanobot signal and background noise.

**Fig. 3:**
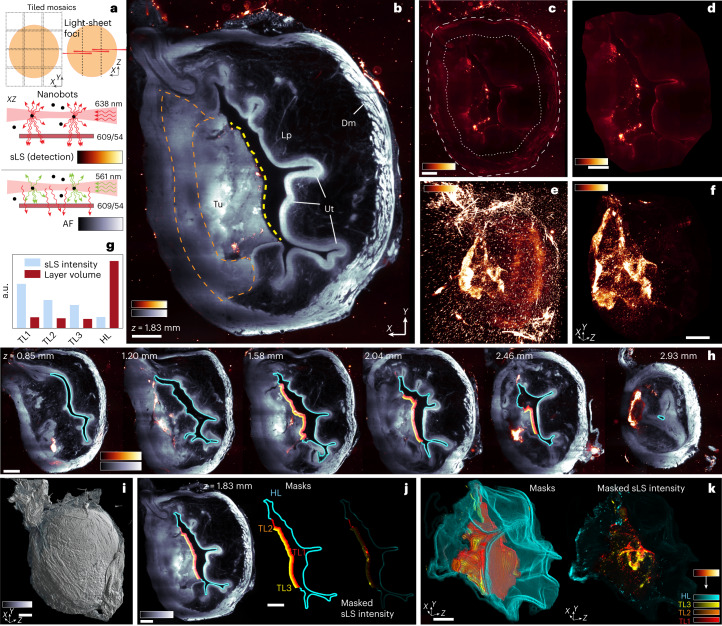
Nanobots penetrate the bladder tumour. **a**, Tiled acquisition layout in *XY* and *XZ* showing the centred light-sheet waist for each *XZ* column; sLS principle where laser light is scattered by particles (for example, nanobots) and passes a filter for detection versus autofluorescence (AF) where the laser is blocked. **b**, Selected plane in bladder centre with tumour; light-sheet excitation from right. Tumour (Tu) detectable with a dashed yellow line delineating its surface exposed to the bladder inner cavity. Healthy tissue defined with a dark lamina propria (Lp) and a bright layer of urothelium (Ut), surrounded by detrusor muscles (Dm). Delineating tumour against inner tissues is more challenging; the tumour’s limits could lie anywhere within the orange dashed area (Supplementary Video 3). *n* = 1. ‘Glow’ colour scale: sLS showing nanobots (inside bladder) and agarose crystals (outside). **c**, sLS signal only, showing nanobots inside bladder, agarose crystals outside and scattered signal in the periphery (muscle). The dashed line shows external tissue boundaries; the dotted line shows the same region after 500 µm digital erosion. **d**, Area inside dotted line in **c**. **e**, Maximum-intensity projection (MIP) of sLS signal, where all external scatterers (agarose, muscle) contribute to signal. **f**, MIP of scattered signal from volume shown in **d**, containing only nanobots. **g**, Integrated sLS signal intensity normalized by layer volume inside four masks shown in **h**,**j**,**k** (Supplementary Methods). TL, tumour layer; HL, healthy layer. a.u., arbitrary units. **h**, Optical sections at different depths with annotations of tissue layers for quantification (**j**). **i**, 3D surface render of bladder, same orientation as **e**,**f**. **j**, Left: tumour and healthy layers of tissues along bladder cavity, detected and annotated (masks) in three dimensions. Cyan, urothelium; HL, healthy tissue; red, tumour surface at 0–33 µm depth; orange, 34–67 µm depth; yellow, 68–100 µm depth. Right: sLS signal coloured with masks. **k**, Left: 3D renders of masks from **h**,**j**. Right: 3D MIP of sLS signal inside masks. Scale bars: **b**–**d**,**i**,**k**, 400 µm; **f**,**h**,**j**, 500 µm. Source data

Next, we applied label-free sLS to detect and localize nanobots in a tumoral bladder with high spatial specificity and to quantify their tumour penetration (Fig. 3). However, while coarse tumour outlines could be easily obtained via autofluorescence (Fig. 3b,h), a more accurate segmentation of its 3D contours proved challenging. Our analysis, therefore, focused on the outer tumour surface that is exposed to the bladder cavity (or lumen), where intensity contrasts with the cavity and visual differences from the opposite and healthy urothelium make it easy to distinguish and annotate (yellow dashed line in Fig. 3b). We segmented three concentric tumour layers (Fig. 3h,j,k and Supplementary Information), starting from the lumen and moving inwards, to estimate the numbers of nanobots at different depths from the tumour surface. We also segmented the exposed healthy tissue layer to compare nanobot penetration therein. The results showed that nanobots accumulated more prominently at the tumour surface, where numbers were about four times higher than at the healthy urothelium (Fig. 3f–k and Supplementary Video 3). Tumour penetration was notable, although nanobot-induced intensity decreased with increasing depth (by about 50% from the first to the third layer) (Fig. 3g). We also attempted an sLS analysis throughout the tumour, using a more subjective 3D annotation of the extent of tumour lesions. This in toto quantification yielded the median penetration depth and occupancy rates in tumoral versus healthy urothelium, confirming prominent nanobot accumulation and penetration in the tumour (Supplementary Fig. 10 and Supplementary Information).

**Fig. 4:**
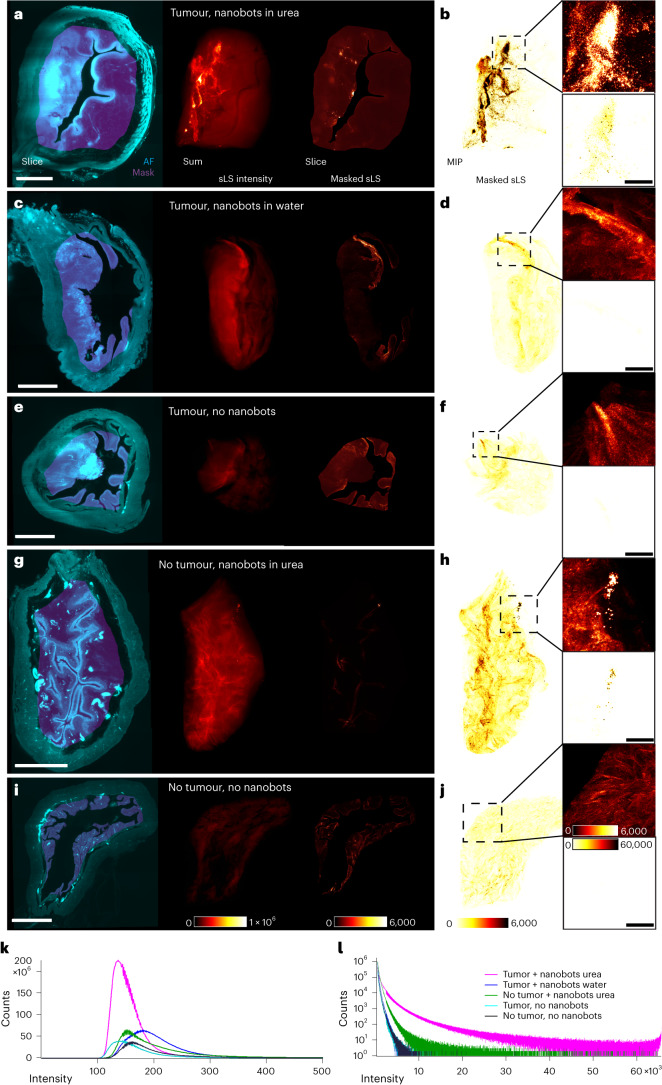
Comparing nanobot retention in bladder tissues with tumours. **a**–**j**, Each tissue undergoes identical processing and visualization (*n* = 1). Samples are imaged in autofluorescence and with V-LS (vertically-polarized light-sheet) and V-CAM (vertically-polarized detection) sLS. Left to right: single slice in mid-plane with autofluorescence (cyan) and processed mask (magenta) of internal tissue after removing internal lumen and digitally eroding an external layer of 500 µm from the outer edge of the bladder; summed intensity of sLS signal inside the mask; single slice of sLS signal inside the mask; MIP of sLS signal inside the mask and two insets showing the same sLS signal with different look-up tables and intensity scales (**j**). Rows represent different animals and conditions. **a**,**b**, Tumour in bladder with nanobots in urea (same sample as in Fig. 3 and Supplementary Fig. 5a–f). **c**,**d**, Tumour in bladder with nanobots in water, that is, ‘without fuel’. **e**,**f**, Tumour in bladder without nanobots (same sample as Supplementary Fig. 5h–m). **g**,**h**, Healthy bladder without tumour and with nanobots in urea. **i**,**j**, Healthy bladder without tumour and without nanobots (same sample as in Supplementary Fig. 4o–x). **k**,**l**, Voxel intensity histograms (3D) of the masked sLS volumes from **a**,**c**,**e**,**g**,**i** (that is, summed intensity inside masks) showing the distribution of the scattered signal on linear (**k**) and logarithmic (**l**) (16-bit) axes. While background distributions in **l** vary for different mask volumes, pink and green curves (nanobots in urea) correlate with MIP images in **a**,**b**,**g**,**h**, with a long high-intensity tail indicating a prominent signal; in other conditions (without nanobots or with nanobots in water) intensity drops off quickly with no counts above intensities of about 7,000. In **g**, the cavity inside the bladder was too thin and collapsed to be segmented, hence the mask includes the full volume (including the lumen), although residual cavity background does not contribute to the high intensities. Look-up table scales (‘Red Hot’ and ‘Inverted Red Hot’) and intensity limits shown in **i**,**j** apply to all panels. Scale bars, **a**,**c**,**e**,**g**,**i**, 1 mm; **b**,**d**,**f**,**h**,**j** insets, 200 µm. Source data

**Fig. 5:**
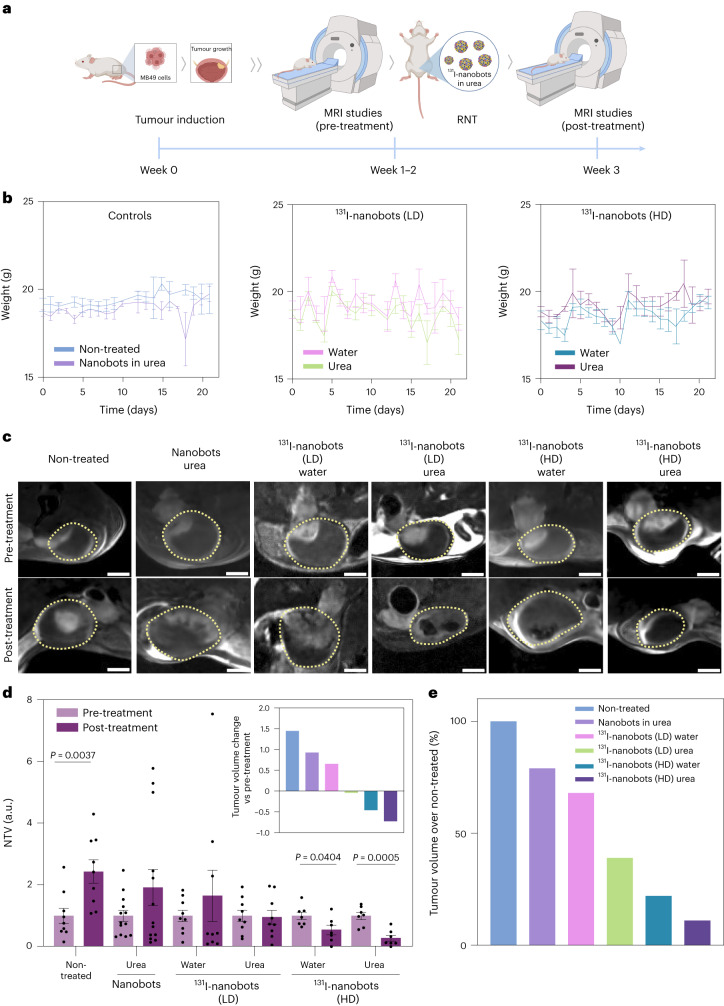
RNT studies using ^131^I-nanobots in a bladder cancer orthotopic murine model. **a**, Schematic representation and timeline of the RNT studies. **b**, Changes in body weight over time, showing mean and s.e.m. (*n* = 9 per group, except *n* = 13 for nanobots in urea and *n* = 7 for high-dose (HD) ^131^I-nanobots in water and urea, biological replicates). LD, low-dose. **c**, DW-MRI 2D slices through the bladder of tumour-bearing mice before and after treatment with radionuclides; low-dose (LD) and high-dose (HD) denote 1.85 MBq and 18.5 MBq doses of ^131^I, respectively; yellow dotted lines show the bladders. Scale bars, 2 mm. **d**, NTV obtained by MRI before and after treatment. Tumour volumes were normalized by the means of the pretreatment values of each group (*n* = 9 per group, except *n* = 13 for nanobots in urea and *n* = 7 for high-dose ^131^I-nanobots in water and urea, biological replicates). Data are presented as mean values and error bars represent the s.e.m. Statistical significances are based on a two-tailed unpaired *t*-test. Inset: tumour volume changes with respect to pretreatment (see **e** for legend). **e**, Post-treatment tumour volumes normalized to control condition (non-treated group). Panel **a** created with BioRender.com. Source data

To validate our interpretations of the PET-CT and MRI images, we analysed the sLS signal in cleared bladders in five conditions: (1) with tumour and nanobots in urea (Fig. 4a,b), (2) with tumour and nanobots in water (Fig. 4c,d), (3) with tumour and without nanobots (Fig. 4e,f), (4) without tumour and with nanobots in urea (Fig. 4g,h) and (5) without either tumour or nanobots (Fig. 4i,j). Comparisons of the total 3D intensity within the internal bladder tissues (Fig. 4k,l; see Supplementary Information for details) clearly showed a prominent sLS signal when nanobots are present and in urea (conditions 1 and 4). Without nanobots or with nanobots in water (conditions 2, 3 and 5) the signal intensity is equally low, and hence identified as background. Results confirmed that, unlike nanobots in water, those in urea successfully self-propel within mouse bladder and reach the tumour site in significant numbers, where they accumulate and penetrate tumour tissues.

The improved accumulation and tumour penetration of active nanobots observed in our experiments could be favoured by alterations in the bladder’s permeability barrier during disease. The urothelium consists of basal, intermediate and superficial cells. Superficial cells, also called umbrella cells, are connected by tight junctions that typically block harmful substance diffusion^46,47^. However, conditions such as urothelial cancer can increase urothelium permeability by reducing or eliminating tight junctions, enabling better particle penetration^48,49^. Moreover, nanobots can degrade the extracellular matrix of the tumour (Supplementary Fig. 11) by locally increasing pH due to the enzymatic reaction^50^ that produces ammonia, potentially resulting in enhanced penetration when compared with their passive counterparts.

## RNT using ^131^I-nanobots

Next, we took advantage of the tumour accumulation properties of nanobots to evaluate their therapeutic effect. Iodine-131 (^131^I) is one of the most common radioisotopes in RNT due to its favourable properties: long half-life (8.01 days) and emission of β particles (maximum energy 0.61 MeV) with a penetration range of 0.8 mm in tissue. Previously^33^, we described the efficient ^124^I-labelling of nanobots by radionuclide absorption on AuNPs present on their surface. Here, we successfully achieved ^131^I-radiolabelling (Fig. 1a) at high radiochemical yields (73 ± 10%) and excellent radiochemical purity (≥99%). The labelled nanobots showed good stability at 37 °C in both water and 300 mM urea for 1 h (Supplementary Fig. 12), and no decrease in the enzymatic activity (Supplementary Fig. 1).

For the in vivo assessment of RNT efficacy, we employed the same murine model and MRI protocol to distribute animals among experimental groups, and nanobots were intravesically administered following the scheme in Table 2 (Fig. 5a).

**Table 2:** Summary of the different experimental scenarios performed in the RNT study

Group	*n*^a^	Compound	Activity (MBq)
1	9	Non-treated	0
2	13	Nanobots in urea	0
3	9	^131^I-nanobots (LD) in water	1.85
4	9	^131^I-nanobots (LD) in urea	1.85
5	7	^131^I-nanobots (HD) in water	18.5
6	7	^131^I-nanobots (HD) in urea	18.5

^a^Experiments were performed in two batches. The first batch included groups 1–4. The second batch included groups 5 and 6. Ten animals were inoculated per group; the ones listed in the table are those that showed tumour growth and were included in the study. In batch 2, five additional animals were included in group 2 as controls, and the results were pooled.

Animals in all groups received an intravesical injection of the corresponding treatment into their empty bladders, leaving the nanobot-containing solution to incubate for 1 h. Then, bladders were emptied, and animals left to recover from anaesthesia. No animal exhibited any apparent radiation-related side effects or notable weight changes during the week following treatment (Fig. 5b). MRI images obtained before treatment showed the presence of a tumour in the upper part of the bladder (Fig. 5c). Administering ^131^I-nanobots with a high dose of ^131^I (18.5 MBq) led to an effective tumour reduction in both urea and water. If ^131^I-nanobots were administered with a low dose of ^131^I (1.85 MBq) the tumour size increased slightly with water and remained constant in urea, suggesting a halt in tumour growth.

Normalization of tumour volume to initial values clearly shows an increase in tumour size (about 2.45 normalized tumour volume, NTV) over time for non-treated animals (Fig. 5d). A similar trend was seen in animals instilled with non-labelled nanobots in 300 mM urea solution (about 1.93 NTV), confirming that nanobots alone do not have therapeutic properties. When animals were treated with lose-dose ^131^I-nanobots in water we also saw a slight increase in tumour size over time (about 1.66 NTV), while injecting low-dose ^131^I-nanobots in urea arrested tumour growth (0.96 NTV). At high doses, ^131^I- nanobots in both media (water and urea) resulted in a significant reduction in tumour size after treatment (about 0.54 and 0.27 NTV, respectively) (Fig. 5d, inset). The RNT effect was more pronounced for high-dose ^131^I-nanobots administered in urea, with a nearly 90% reduction in tumour volume with respect to non-treated animals (Fig. 5e).

In summary, nanobots labelled even with low doses of ^131^I yield a net tumour volume reduction as their active motion leads to higher tumour accumulation (Fig. 5d, inset). This efficacy at low doses should facilitate the translation of nanobots with therapeutic radionuclides to clinical settings. High doses of ^131^I-nanobots led to a more pronounced reduction in tumour volume and animals were able to maintain their weight within standard ranges, suggesting no severe adverse effects.

## Conclusions

In this study, we demonstrated the successful tumour accumulation of urease-powered nanobots in an orthotopic murine model of bladder cancer following intravesical injection, utilizing various complementary imaging techniques. PET combined with MRI revealed an increased macroscopic accumulation of radiolabelled nanobots with self-propelling capacity within the tumour. These results were validated by ICP-MS. Additionally, polarization control in sLS imaging confirmed the presence of nanobots throughout the entire bladder, enhancing their optical response into clearly visible foci that stood out against the tissue background signal. These findings position sLS as a valuable validation tool for in vivo observations, providing microscopic information due to its superior 3D resolution. Moreover, the motion-enhanced tumour accumulation enables radiotherapeutic self-propelled nanobots to have an effect at doses substantially lower than those required for efficient passive particles. This suggests that ^131^I-carrying nanobots can effectively treat bladder tumours in confined spaces, presenting an alternative treatment for scenarios where traditional therapeutic approaches, such as BCG, routinely fail. Overall, these results provide clear evidence of the potential use of urease-powered nanobots as efficient therapeutic carriers for bladder cancer therapy.

## Methods

### Nanobot synthesis

Nanobots were prepared as previously described^33^. In brief, MSNPs were synthesized using a modified Stöber method^41^, reacting triethanolamine (35 g), ultrapure water (20 ml) and hexadecyltrimethylammonium bromide (CTAB; 570 mg) at 95 °C for 30 min while stirring. Tetraethyl orthosilicate (1.5 ml) was subsequently added dropwise; the mixture was left to react for 2 h at 95 °C and the resulting MSNPs collected by centrifugation and washed in ethanol (three times, 2,500*g*, 5 min). To remove the CTAB template, MSNPs were placed under reflux in acidic methanol (1.8 ml HCl, 30 ml methanol) for 24 h. Then, MSNPs were collected by centrifugation and washed three times in ethanol (2,500*g*, 5 min) before incorporating amine modification by adding APTES (6 µl per mg of MSNP) to MSNPs (1 mg ml^−1^) in a 70% ethanolic solution at 70 °C, stirring vigorously for 1 h. MSNPs-NH_2_ were collected and washed three times in ethanol and three times in water by centrifugation (three times, 1,150*g*, 5 min). MSNPs-NH_2_ were resuspended in PBS at a concentration of 1 mg ml^−1^ and total volume of 900 µl, and activated with glutaraldehyde (100 µl) for 2.5 h at room temperature. The activated MSNPs-NH_2_ were collected and washed in PBS three times by centrifugation (1,150*g*, 5 min), resuspended in a solution of urease (3 mg ml^−1^) and heterobifunctional PEG (1 μg PEG per mg of 5 kDa HS-MSNPs-NH_2_) in PBS, and reacted for 24 h at room temperature. The resulting nanobots were then collected and washed three times in PBS by centrifugation (1,150*g*, 5 min) before resuspending them in a dispersion of AuNPs, prepared as previously described^51^, leaving them react for 10 min, and thoroughly washing by centrifugation (three times, 1,150*g*, 5 min).

Hydrodynamic size distribution and surface charge of the MSNPs, MSNPs-NH_2_, nanobots and AuNP-decorated nanobots were determined using a Wyatt Mobius dynamic light scattering system and a Malvern Zetasizer, respectively. In all cases, concentration was 20 µg ml^−1^ and acquisition time 5 s, using three runs per experiment. Three measurements per particle type were performed.

### Synthesis of FITC MSNPs

A mixture of FITC (2 mg), ethanol (5 ml) and APTES (400 µl) was prepared and stirred for 30 min. Then, the previously described protocol for MSNP synthesis was followed, except that we added tetraethyl orthosilicate (1.25 ml) dropwise in combination with the FITC–APTES mixture (250 µl). The functionalization steps to obtain FITC-labelled nanobots were as aforementioned.

### Synthesis of AuNPs

AuNPs were synthesized using a reported method^33^. In brief, all materials were cleaned using freshly prepared aqua regia, thoroughly rinsed with water, and air-dried. Subsequently, a 1 mM AuCl_4_ solution was heated to its boiling point while stirring in a round-bottom flask integrated into a reflux system. Following this, 10 ml of sodium citrate solution (30.8 mM) was added, and the solution was boiled for 20 min, resulting in a red colour. The solution was then allowed to cool to room temperature while stirring for 1 h. The resulting AuNPs were stored in the dark and characterization was conducted using transmission electron microscopy.

### Enzymatic activity

Enzymatic activity of nanobots, ^18^F-nanobots and ^131^I-nanobots was measured using phenol red. To do so, 2 µl of nanobots (1 mg ml^−1^) were added to a 96-well plate and mixed with 200 µl of different urea solutions (0, 50, 100, 200 mM) in 1.1 mM phenol red. Absorbance at 560 nm was measured over time at 37 °C.

### Nanobot motion dynamics through optical microscopy

Optical videos of nanobots were acquired using a Leica Thunder microscope, coupled with a Hamamatsu high-speed CCD camera and a ×1.25 objective. For this, the nanobots were centrifuged and resuspended in 50 µl of PBS (final concentration of 20 mg ml^−1^). Then, a Petri dish was filled with 3 ml of either PBS or a 300 mM solution of urea (in PBS) and observed under the microscope. A 5 µl drop with nanobots (20 mg ml^−1^) was then added to the liquid-filled Petri dish and videos were recorded at 25 frames per second. Video pixel intensity distributions in ROIs were analysed at 15 s intervals using ImageJ software.

### Radiolabelling of nanobots with [^18^F]F-PyTFP

#### Synthesis of [^18^F]F-PyTFP

[^18^F]F-PyTFP was synthesized in a Neptis xSeed module (Optimized Radiochemical Applications), following a previously reported method^33^.

#### Synthesis of ^18^F-labelled nanobots

Nanobots were labelled with [^18^F]F-PyTFP, on the basis of a previously established procedure with minor modifications^33^. In brief, 200 µl of nanobot solution (1 mg ml^−1^) was centrifuged (10 min, 13,853*g*), resuspended in 10 µl of PBS (1 mM, pH 8), and incubated with 4 µl of [^18^F]F-PyTFP in acetonitrile (about 37 MBq) for 35 min at room temperature. After incubation, the reaction mixture was diluted with water (200 µl) and purified by centrifugation (5 min, 13,853*g*). The resulting pellet was then rinsed three times with water before being measured in a dose calibrator (CPCRC-25R, Capintec). Radiochemical yield was calculated as the ratio between the amount of radioactivity present in the nanobots after washing and the initial amount of radioactivity. Radiochemical purity after purification was ≥99%, as determined by radio thin-layer chromatography (radio-TLC) using iTLC-SG chromatography paper (Agilent Technologies) and dichloromethane and methanol (2:1) as the stationary and mobile phases, respectively. TLC plates were analysed using a TLC reader (MiniGITA, Raytest).

### Stability of ^18^F-nanobots

The stability of ^18^F-labelled nanobots was determined using the following media: (1) 300 mM urea, (2) water, and (3) urine from tumour-bearing animals. ^18^F-labelled nanobots (10 µl) were incubated with the corresponding solution (100 µl) for 1 h at room temperature. Then, nanobots and supernatant were separated by centrifugation and collected, and radioactivity measured in a dose calibrator (CPCRC-25R).

### Radiolabelling of nanobots with ^131^I

The radioiodination of urease nanobots was performed by incubating nanobots with injectable [^131^I]NaI solution (925 MBq ml^−1^; GE HealthCare). In brief, 400 µl of urease nanobot solution (1 mg ml^−1^) was centrifuged (13,853*g*, 5 min), resuspended in 100 µl of PBS (10 mM, pH 7.4) and incubated with 25 µl or 185 µl of injectable [^131^I]NaI (about 42.55 or 277.5 MBq, respectively) for 30 min, depending on the desired final activity. After incubation, the reaction mixture was purified by centrifugation (13,853*g*, 5 min). The resulting precipitate was washed three times with water (100 µl). Radioactivity in the supernatant and precipitate was determined using a dose calibrator (CPCRC-25R), and both fractions were analysed by radio-TLC, as for ^18^F-nanobots.

### Animal model development

Mice were maintained and handled in accordance with European Council Directive 2010/63/UE and internal guidelines. All experimental procedures were approved by the CIC biomaGUNE ethics committee and local authorities (Diputación Foral de Guipuzcoa, PRO-AE-SS-276). Image analysis (both PET and MRI) was blinded towards group distribution of the animals.

The orthotopic murine model of bladder cancer was generated by intravesical administration of MB49 cells (murine carcinoma bladder cell line) to C57BL/6JRj female mice (8 weeks old, Janvier). For experiments aimed at determining tumour accumulation (four groups; details below), six animals were inoculated per group, as determined using precision analysis, with the following assumptions: required precision, 20%; expected s.d., ±20%; confidence, 95%; animal loss, 20%. For therapeutic efficacy experiments (six groups; details below), ten animals were included per group, as calculated using a one-tailed Student *t*-test, difference between two independent means, with the following assumptions: null hypothesis, treatment does not affect tumour growth; *α*, 0.05; 1 − *β*, 0.95; s.d., ±50%; expected differences between groups, 50%; animal loss, 20%. As the experiment was conducted in two batches for operational reasons, one control group was included in both batches (Table 2), and then all animals were pooled. For tumour establishment, mice were anaesthetized by inhalation of 3% isoflurane in pure O_2_ and maintained by 1.0–1.5% isoflurane in 100% O_2_. Then, the bladder was emptied, and chemical lesions induced on the urothelium by intravesically instilling 50 µl of poly-l-lysine (Sigma-Aldrich) through a 24-gauge catheter for 15 min. Subsequently, the bladder was emptied again and MB49 cells (10^5^ cells) in high-glucose DMEM (100 µl) were instilled for 1 h before removing the catheter and emptying the bladder via abdominal massage. Throughout the experiments, mice were monitored and weighed for health and welfare monitoring. A human endpoint was applied if weight loss exceeded 20% or on the basis of clinical symptoms, under the criteria of the veterinarian in charge.

### Tumour size tracking

MRI studies were conducted 7 and 14 days after tumour induction, using a 7 T Bruker BioSpec USR 70/30 scanner (Bruker BioSpin) equipped with a BGA-12S gradient insert of 440 mT m^−1^ and a 112/086 QSN resonator (T12053V3) for radiofrequency^14^ transmission, and a rat brain surface coil (T11205V3) for RF reception (both operating at 300 MHz). Animals were anaesthetized with isoflurane (4% for induction and 1.5% for maintenance in a 50% O_2_/50% N_2_ mixture) and placed on an MR-compatible cradle. Body temperature and respiration rate were continuously monitored using an MR-compatible monitoring device (model 1030 SA, Small Animal Instruments), interfaced to a small-rodent air heater system to maintain body temperature. After acquiring reference images, a spin-echo-based diffusion-weighted imaging sequence was used to image tumours, using the following parameters: echo time (*T*_E_) = 22.3 ms, repetition time (*T*_R_) = 2,500 ms, *n* = 2 averages, one A0 image (basal image with *b* = 0 s mm^−2^) and one DW image acquired using diffusion gradients in the (1, 0, 0) direction with a gradient duration *δ* = 4.5 ms and a gradient separation *Δ* = 10.6 ms, giving *b* = 650 s mm^−2^, a 16 × 16 mm^2^ field of view, image matrix size of 160 × 160 points, 20 consecutive slices of 0.5 mm thickness (no gap, acquired in interleaved mode) and a bandwidth of 192.9 Hz per pixel. To visualize tumours, images were postprocessed with ImageJ software, dividing images acquired with a diffusion gradient (*b* = 650 s mm^−2^) by those acquired without (*b* = 0 s mm^−2^), and applying a 3D Gaussian filter (*σ*_*x*_ = *σ*_*y*_ = *σ*_*z*_ = 0.7) to the result. Tumours were manually delineated to determine their volume.

### In vivo biodistribution

On day 15 after tumour induction, mice were randomized into four groups to obtain homogeneous average tumour volume distributions among groups. PET-CT scans (MOLECUBES β and X-CUBE scanners) were acquired 3 h after intravesically administering 100 µl of ^18^F-BSA (groups 1 and 2) or ^18^F-urease (groups 3 and 4) nanobots at a concentration of 200 µg ml^−1^, using either water (groups 1 and 3) or 300 mM urea in water (groups 2 and 4) as vehicle (Table 1). For image acquisition, animals were induced with anaesthesia (5% isoflurane in pure oxygen) and placed in a supine position before massaging the abdominal region for bladder evacuation. Immediately afterwards, the corresponding ^18^F-labelled nanobots (^18^F-BSA/^18^F-urease in water/urea) were instilled in the bladder through a 24-gauge catheter and incubated for 1 h, before removing the catheter, emptying the bladder and leaving the mice to recover from anaesthesia. At *t* = 3 h after administration, animals were re-anaesthetized and 10 min static whole-body PET images acquired, followed by CT scans. PET images were reconstructed using the 3D ordered subset expectation maximization reconstruction algorithm with random, scatter and attenuation corrections. PET-CT images of the same mouse were co-registered and analysed using the PMOD image processing tool. Plots of concentration of radioactivity versus time were obtained by creating a volume of interest on the upper bladder region using a 3D contour tool and measuring activity (decay corrected) in kilobecquerels per organ. Results were corrected by applying a calibration factor and then normalized by MRI-derived tumour volume.

### Ex vivo studies

#### Histopathologic analyses

After completing all imaging, selected bladders (*n* = 3 per group) from tumour-bearing and healthy animals were removed in aseptic conditions and immediately fixed in 4% formaldehyde. Then, bladders were embedded in paraffin before taking 2–3 µm sections for haematoxylin–eosin staining. Representative images were obtained from all conditions for histopathologic examination.

#### ICP-MS analysis

Measurements were performed on a Thermo iCAP Q ICP-MS (Thermo Fisher Scientific) coupled with an ASX-560 autosampler (CETAC Tech). After completing all imaging, animals were killed, and selected bladders (*n* = 2 per group; four groups) collected and digested in 1 ml of HNO_3_:HCl (4:1 mixture). The dispersion was boiled until organs were completely dissolved. Then, the solution was cooled to room temperature and analysed using ICP-MS to determine the concentration of Au in each sample, transforming the results into percentages of injected dose per gram of tissue (%ID g^−1^).

#### Immunohistochemistry and confocal microscopy imaging

For immunohistochemistry analyses, tumour-bearing animals received FITC-labelled nanobots in water or 300 mM urea (*n* = 4 per group), as described above, for PET-CT studies. Additionally, tumour-bearing animals without nanobots served as a control group (*n* = 2). In all cases, bladders were collected, frozen and cut into 10 µm sections that were immediately fixed in 10% formaldehyde for 15 min, washed with 10 mM PBS and then incubated in 50 mM NH_4_Cl in PBS for 5 min before rinsing again with PBS. Permeabilization was performed with methanol:acetone (1:1) for 5 min at room temperature and 0.1% Triton in PBS for 5 min. After PBS washing, samples were saturated with a solution of 5% BSA–0.5% Tween in PBS for 15 min at room temperature and incubated for 1 h at room temperature with mouse anti-FITC (1:100, Abcam) in 5% BSA–0.5% Tween. Sections were washed three times with 10 mM PBS for 5 min and incubated for 30 min at room temperature with secondary antibody Alex Fluor 647 donkey anti-mouse IgG (Molecular Probes, Life Technologies, 1:1,000) in 5% BSA–0.5% Tween in PBS, washed again in PBS (3 × 5 min) and mounted with a ProLong antifade kit with 4,6-diamidino-2-phenylindole (DAPI; Molecular Probes, Life Technologies). Images were acquired with a Leica STELLARIS 5 confocal microscope (UPV/EHU Scientific Park) with identical settings for all sections: ×10 magnification with tile imaging and stitching (typically 4 × 5 field of view). Laser line and detection windows were 405 nm and 440–503 nm for DAPI, 489 nm and 494–602 nm for FITC white laser and 653 nm and 660–836 nm for Alexa647 white laser.

### Optical clearing

After perfusion with 4% paraformaldehyde and PBS, bladder samples were removed and further fixed in 4% paraformaldehyde overnight at 4 °C, then embedded in a 5 ml syringe with 0.8% low-melting-point agarose to form a cylindrical block and enable easy mounting in the quartz cuvette. The entire block was progressively dehydrated using methanol:H_2_O at 4 °C (30%:70% for 1 h, 50%:50% for 1 h, 70%:30% for 1 h, 100%:0% for 1 h, then 100% methanol overnight and again for 4 h) and finally immersed in benzyl alcohol–benzyl benzoate (BABB) as refractive index matching solution for imaging. For in vitro comparisons of green FITC nanobots with commercial red particles, we used DiagNano (Creative Diagnostics) red fluorescent silica nanoparticles, 1 µm diameter, resistant to BABB clearing.

### Autofluorescence and polarized sLS imaging

Light-sheet imaging was performed on MacroSPIM, a custom system for cleared whole-organ imaging developed at IRB Barcelona^44,45^. In brief, samples are embedded in an agarose block, cleared together with the sample and imaged inside a quartz cuvette. Autofluorescence imaging used lasers at 488, 561 or 638 nm delivering illumination through a 50 mm achromatic doublet cylindrical lens (ACY254-050-A, Thorlabs). To reduce stripe artefacts, the light sheet is pivoted with a resonant scanner SC-10 (EOPC) along a 4f telescope with G322288322 100 mm achromatic doublet lenses (QI Optic Photonics). Tissue autofluorescence is collected through band- or long-pass fluorescence filters and recorded with an ORCA Flash v2 camera (Hamamatsu Photonics). Imaging was performed at ×9.6 with a ×8 zoom, ×2 lens and ×0.6 tube lens. The light sheet was flattened across the field of view, yielding 5–6 µm of axial resolution. 3D imaging was done in steps of 2.5 µm. Whole-bladder imaging was performed in 2 × 3 or 3 × 4 *XY* tiles, depending on organ size.

sLS imaging was achieved by removing the fluorescence filter or using any filter transmitting the laser. Light-sheet pivoting reduced laser speckle noise, resulting in temporal averaging of laser coherence as shown earlier^52^. The orientation of linear light-sheet polarization in illumination was controlled by rotating a half-wave plate (AHWP05M-600, Thorlabs) before the pivot scanner. The detected signal was selected in polarization using a rotating linear polarizer (LPVISC100, Thorlabs) before the filter wheel in detection, introducing >50% intensity loss in fluorescence detection. While sLS signal distribution in general changes with the polarizer’s orientation, the tissue autofluorescence signal remains unaffected by the polarizer’s rotation. sLS yields a spatial resolution of 2.4 ± 0.3 µm in BABB, which is comparable to the resolution in fluorescence light-sheet imaging (confirmed by fitting a Gaussian function to the *XY* image response of a single particle, Supplementary Fig. 8l–m) and close to the theoretical resolution in air (1.53 µm with numerical aperture (NA) = 0.2 at maximum macro zoom ×8).

### Image processing and 3D analysis

Image processing, segmentation and analysis of light-sheet datasets was done with ImageJ/Fiji, while Figs. 3 and 4 were generated with Imaris Viewer 9.9 (https://imaris.oxinst.com/imaris-viewer) and Supplementary Video 3 was generated with Imaris 9 (https://imaris.oxinst.com/) (Bitplane, Oxford Instruments). Tiled light-sheet datasets were stitched with MosaicExplorerJ^53^. Bladder tissue 3D segmentation was performed using custom ImageJ/Fiji macros for semi-automated 3D annotation of large volumes in virtual mode. In brief, a first script, ‘Macro1’, loads 3D image stacks, enables user annotation of ROIs in several planes and automatically interpolates the ROIs to generate and export 3D masks. ROIs were drawn every 15 planes (every 37.5 µm) to facilitate good segmentation continuity while keeping annotations to a reasonable minimum. A second script, ‘Macro2’, performs the mathematical or Boolean operations, plane by plane without loading the entire stacks into memory, either between 3D masks or between a 3D mask and the original data, saving the result as a new stack. All masks were generated by annotating autofluorescence images.

Both tumour and healthy tissue surface layers (Fig. 3) were delineated using Fiji’s wand and lasso tools on the bladder cavity in a mask. Calling this first iteration BC1, subsequent runs of Macro1 then automatically dilate this 3D contour by a defined pixel amount to yield new mask iterations, BC2, BC3 and so on, with increasing dilations. The first layer containing both tumour and healthy tissue, mask L1, is obtained by subtracting mask BC1 from BC2 and so forth, yielding L2 and L3 as concentric layers. The tumour volume closest to the cavity was obtained by annotating the tumour with wand and lasso tools to create a mask T1, while the healthy urothelium 3D layer was detected separately into mask U1. Subtracting U1 from L1 yields the surface layer of the tumour, and so forth: L2 − U1, L3 − U1. Conversely, the first layer of the urothelium is obtained by subtracting T1 from L1. All layers in Fig. 3 were defined to have 33 µm thickness.

The same suite of macros and procedures (ImageJ wand tool, digital erosion of 500 µm and so on) were used to delineate and segment the inner part of the bladder tissue and then estimate the bladder’s internal tissue volume (Fig. 4, see above for details). Histograms of the scattered signal intensity were created in Fiji by combining the scattered signal and mask.

### RNT using ^131^I-nanobots

Between days 8 and 15 after tumour implantation, animals were divided into six groups (groups 1–6), trying to achieve similar average tumour volumes across groups (Table 2). For the experiments, animals were induced with anaesthesia (5% isoflurane in pure O_2_) and positioned supine before emptying the bladder by massaging the abdominal region. Immediately afterwards, 100 µl of the appropriate treatment at a concentration of 400 µg ml^−1^ (Table 2) was instilled into the bladder using a 24-gauge catheter. Treatment and vehicle (water or urea) remained in the bladder for 1 h before removing the catheter. The bladder was emptied again by abdominal massage and mice recovered from anaesthesia in their cages, replacing animal cage sawdust 24 h after treatment to remove radioactive contamination.

### Therapeutic efficacy determined by MRI

Two MRI studies were performed on each mouse: (1) between days 7 and 14 after tumour inoculation to randomize animals among groups and measure initial (pretreatment) tumour volumes; (2) between days 16 and 21 after tumour inoculation (post-treatment) to evaluate therapeutic efficacy. MRI was conducted using 7 T Bruker BioSpec and 11.7 T Bruker BioSpec scanners (both with ParaVision 7 software), depending upon availability. This did not affect the results since the external field is not critical for anatomical imaging^14^. Imaging experiments were conducted using the same imaging parameters and processing as explained above (Tumour size tracking). In the case of the 11.7 T scanner the set-up consisted of a mouse heart surface coil for the reception and a volumetric coil for transmission. Tumour volumes in each slice were determined from manually drawn volumes of interest covering the tumour area.

### Statistical analysis

In PET imaging studies, percentages of injected dose (% ID) and injected dose per tumour volume (% ID cm^−^^3^) were compared using one-way ANOVA. Differences between groups were determined using Tukey’s multiple comparisons test. NTV in RNT section was obtained from a *t*-test of unpaired values. Data distribution was assumed to be normal, but this was not formally tested. Statistical analyses were performed with GraphPad Prism v.8.

### Reporting summary

Further information on research design is available in the Nature Portfolio Reporting Summary linked to this article.

## Online content

Any methods, additional references, Nature Portfolio reporting summaries, source data, extended data, supplementary information, acknowledgements, peer review information; details of author contributions and competing interests; and statements of data and code availability are available at 10.1038/s41565-023-01577-y.

### Supplementary information

Supplementary Information Supplementary Figs. 1–12, Extended Discussion and Methods.

Reporting Summary

Supplementary Video 1 Enhanced diffusion of nanobots in PBS.

Supplementary Video 2 Swarming behaviour of nanobots in the presence of 300 mM urea.

Supplementary Video 3 3D localization of nanobots in urea inside a tumoral bladder, and penetration analysis into the tumour.

Supplementary Video 4 Tracking of tracer particles in the absence and presence of swarms, in both PBS and 300 mM urea.

### Source data

Source Data Fig. 1 Raw numerical data from Fig. 1.

Source Data Fig. 2 Raw numerical data from Fig. 2.

Source Data Fig. 3 Raw numerical data from Fig. 3.

Source Data Fig. 4 Raw numerical data from Fig. 4.

Source Data Fig. 5 Raw numerical data from Fig. 5.

## Data Availability

The unprocessed raw image data from PET, CT, sLS and MRI are available from the authors upon request. Source data are provided with this paper.

## Code availability

Quantification of light-sheet images was performed using custom ImageJ macro scripts that facilitate user-guided segmentation, particularly for datasets exceeding available RAM (typically >500 GB). These macros, developed in the ImageJ Macro language, were executed using Fiji with IJ v.2.3.0/1.53d. The full set of macros is part of a comprehensive suite for ‘Large Image Analysis’ and will be detailed in a separate technical article. Consequently, the code will only be made available upon reasonable request.
