# Supplementary material for: Urease-powered nanobots for radionuclide bladder cancer therapy
Source: Nat Nanotechnol. 2024 Jan 15;19(4):554–64. doi: 10.1038/s41565-023-01577-y (PMC11026160; doi:10.1038/s41565-023-01577-y)
Supplement: Supplementary file 1 — Supplementary Figs. 1–12, Extended Discussion and Methods. [file 41565_2023_1577_MOESM1_ESM.pdf]

nature nanotechnology

Article

https://doi.org/10.1038/s41565-023-01577-y

Urease-powered nanobots for radionuclide bladder cancer therapy

In the format provided by the authors and unedited

Table-of-contents

Supplementary videos:

- **Supplementary Video 1** caption: Enhanced diffusion of nanobots in PBS. The video was recorded at 25 fps with a Hamamatsu high-speed CCD camera and a 1.25X objective.
- **Supplementary Video 2** caption: Swarming behavior of nanobots in the presence of 300 mM urea. The video was recorded at 25 fps with a Hamamatsu high-speed CCD camera and a 1.25X objective.
- **Supplementary Video 3** caption: **3D localization of nanobots in urea inside a tumoral bladder, and penetration analysis into the tumor.**
  The video is a sequence of 8 steps. 0:00-0:03: Rotating view of surface rendering of the bladder followed by Maximum intensity projection (MIP) of scattered signal of the entire sample; 0:04-0:15: slice per slice view of autofluorescence (Bone LUT) and scattered signal (Glow LUT); 0:16-0:21: slice per slice view of nanobots scattered signal (Glow LUT) inside a masked volume that exclude a 500um layer to exclude agarose and muscles; 0:16-0:28: Rotating MIP of the previous nanobots signal showing their distribution in the internal parts of the bladder with tumor; 0:29-0:43: slice per slice view of nanobots scattered signal (Glow LUT) and autofluorescence, together with segmented layers (4 colors: yellow, orange and red in tumor, cyan in urothelium) inside which intensity quantification was made; 0:44-0:51: Rotating view of segmented layers (same layers, 4 colors) to show the concentric layers at the surface of the tumor, and the urothelium "healthy" layer inside the bladder cavity; 0:52-0:57: Rotating view of the scattered signal inside the 4 layers; 0:58-1:06: Summary views, medley of the previous.
- **Supplementary Video 4** caption: Tracking of tracer particles in the absence and presence of swarms, both in PBS and 300 mM urea. Videos were recorded at 25 fps with a Hamamatsu high-speed CCD camera and using a 10X objective.

Supplementary Figures:

- **Supplementary Figure S1:** Enzymatic activity of radiolabeled and non-radiolabeled nanobots.
- **Supplementary Figure S2:** Tracking of 2 μm-sized tracer polystyrene particles.
- **Supplementary Figure S3**: Tumor volume evolution over time.

- **Supplementary Figure S4**: Representative histological images of hematoxylin-eosin staining.
- **Supplementary Figure S5**: Radioactivity accumulation.
- **Supplementary Figure S6**: Confocal microscopy in *ex vivo* bladders.
- **Supplementary Figure S7**: Orthogonal views with lightsheet imaging in fluorescence and scattered modes.
- **Supplementary Figure S8**: Polarized Scattered Lightsheet (sLS) microscopy with nanobots in vitro and in healthy bladder tissues.
- **Supplementary Figure S9**: Polarized scattered lightsheet (sLS) microscopy reveals nanobots in bladder tissues with tumors.
- **Supplementary Figure S10**: Full 3D analysis of sLS signal in Tumor vs Urothelium.
- **Supplementary Figure S11**: Extracellular matrix degradation.
- **Supplementary Figure S12**: Thin layer chromatography profiles.

Extended Supplementary Discussion:

- Supplementary discussion on confocal imaging of nanobots in ex-vivo bladders
- Supplementary discussion on polarized scattered light sheet (sLS) microscopy to detect nanobots in ex-vivo bladder tissues.
- Supplementary analysis of sLS signal distribution in whole tumor vs. urothelium.

Supplementary Methods:

- Tracer particle motion dynamics through optical microscopy
- *In toto* tumor analysis of nanobots localization
- Electrical resistance measurements

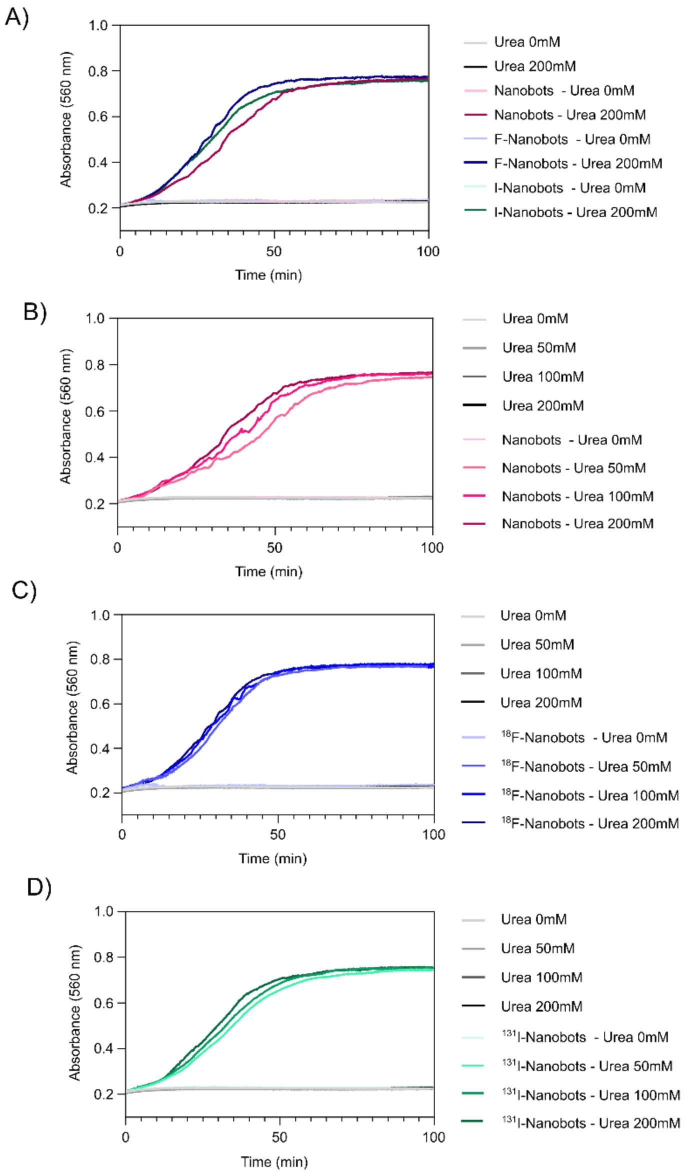

Supplementary Figure S1: Enzymatic activity of radiolabeled and non-radiolabeled nanobots. A) Non-labeled nanobots and nanobots labeled with ^{18}F and ^{131}I in 0 and 200 mM urea (n = 3 per group, technical replicates). B) Nanobots at different concentrations of urea (0, 50, 100 and 200 mM; n = 3 per urea concentration, technical replicates). C) ^{131}I-Nanobots at different concentrations of urea (0, 50, 100 and 200 mM; n = 3 per urea concentration, technical replicates). D) ^{18}F-Nanobots at different concentrations of urea (0, 50, 100 and 200 mM; n = 3 per urea concentration, technical replicates).

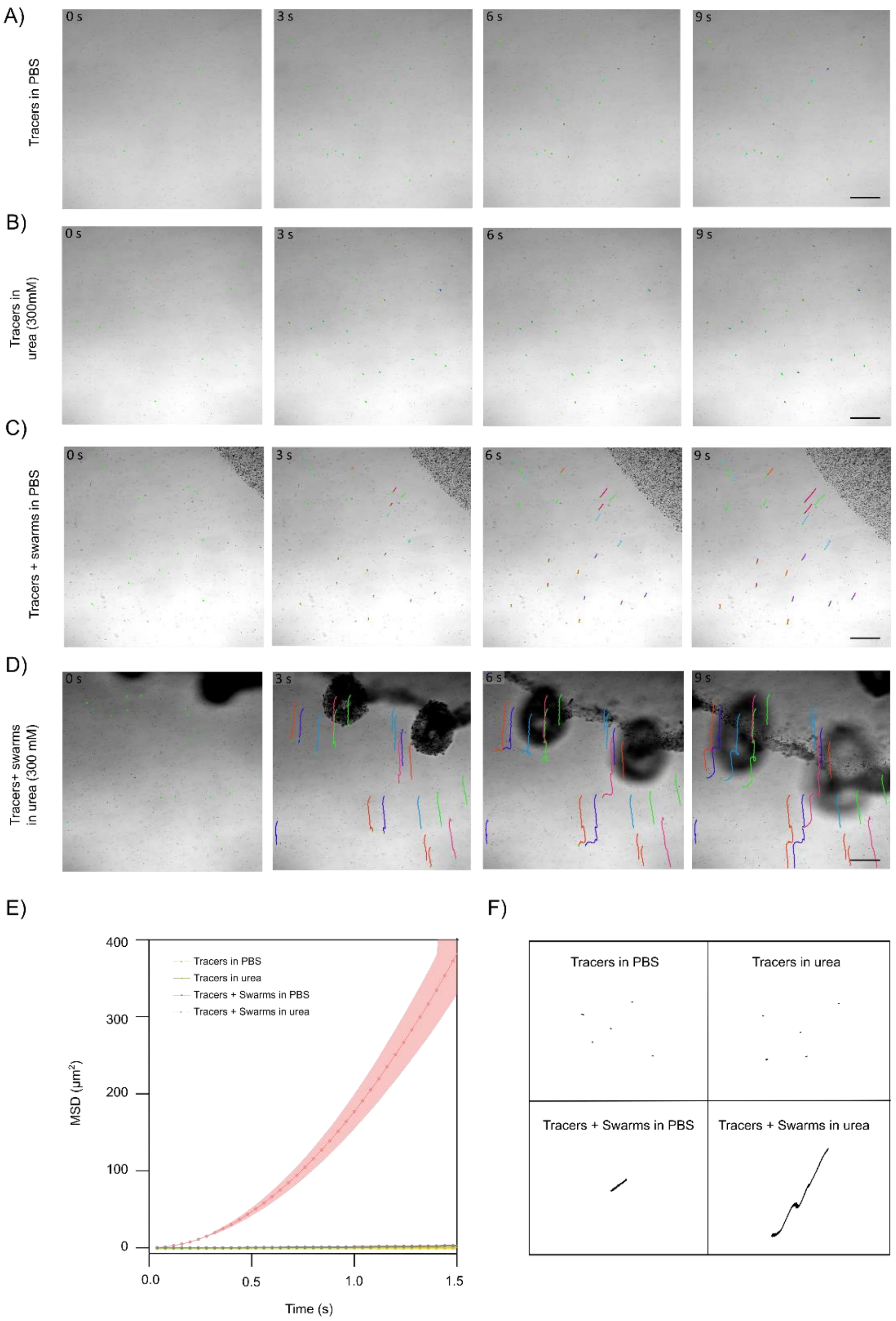

Supplementary Figure S2: Tracking of 2 µm-sized tracer polystyrene particles in A) PBS, B) 300 mM urea, C) in the presence of a swarm of nanobots in PBS, and D) in the presence of a swarm of nanobots in 300 mM urea. The study comprises n=20 technical replicates. The scale bar represents 200 µm. E) Mean-squared displacement (MSD) and F) representative trajectories of the various conditions analyzed (n=20 technical replicates, with error bars from MSD indicating the standard error of the mean).

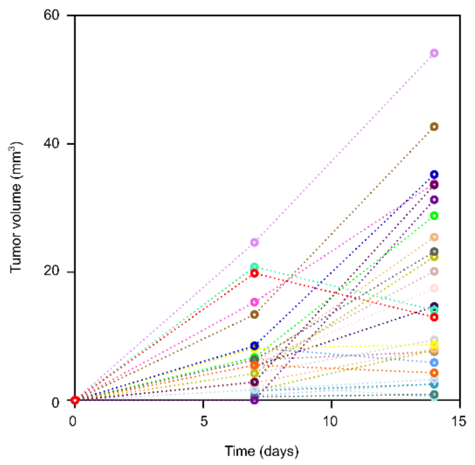

Supplementary Figure S3: **Tumor volume evolution over time** determined by MRI for all animals developing tumor (n=27, biological replicates) at day 7 and 14 from cell implantation. Each color represents an individual animal.

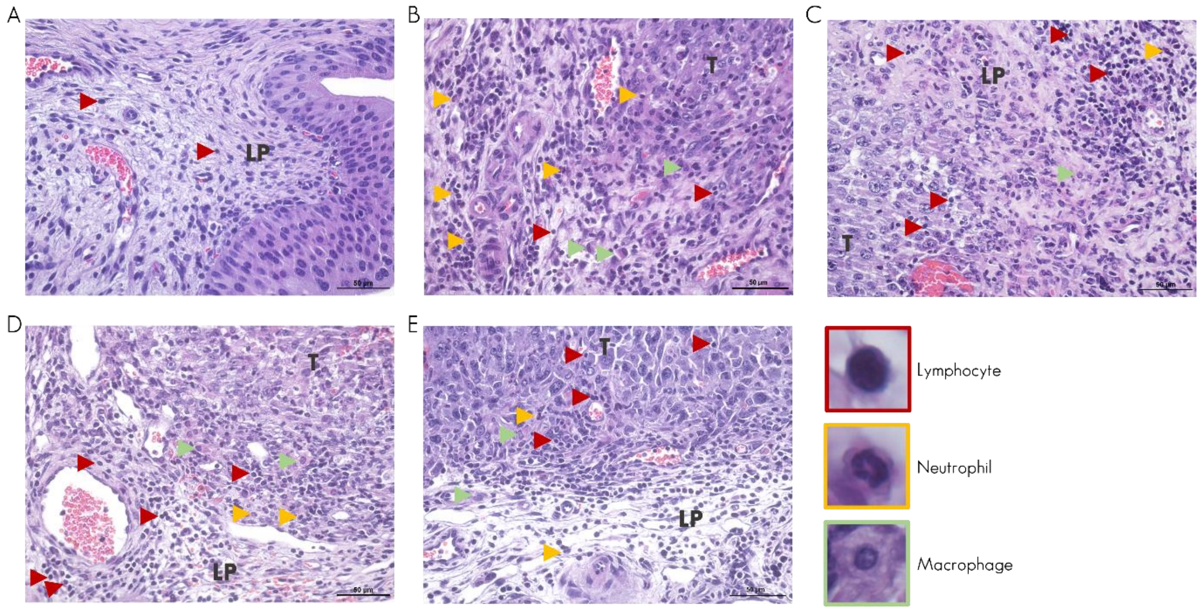

Supplementary Figure S4: Representative histological images of hematoxylin-eosin staining. Bladder sections from representative animals corresponding to: A) Control group (n=2), B) Group 1: BSA-NPs administered with water (n=3), C) Group 2: BSA-NPs administered with a 300 mM urea solution in water (n=2), D) Group 3: Nanobots administered with water (n=3), and E) Group 4: Nanobots administered with a 300 mM urea solution in water (n=3, biological replicates). Red triangles indicate lymphocytes, green triangles represent macrophages, and yellow triangles denote neutrophils. "T" refers to the tumor part in the bladder, and "LP" refers to Lamina Propria.

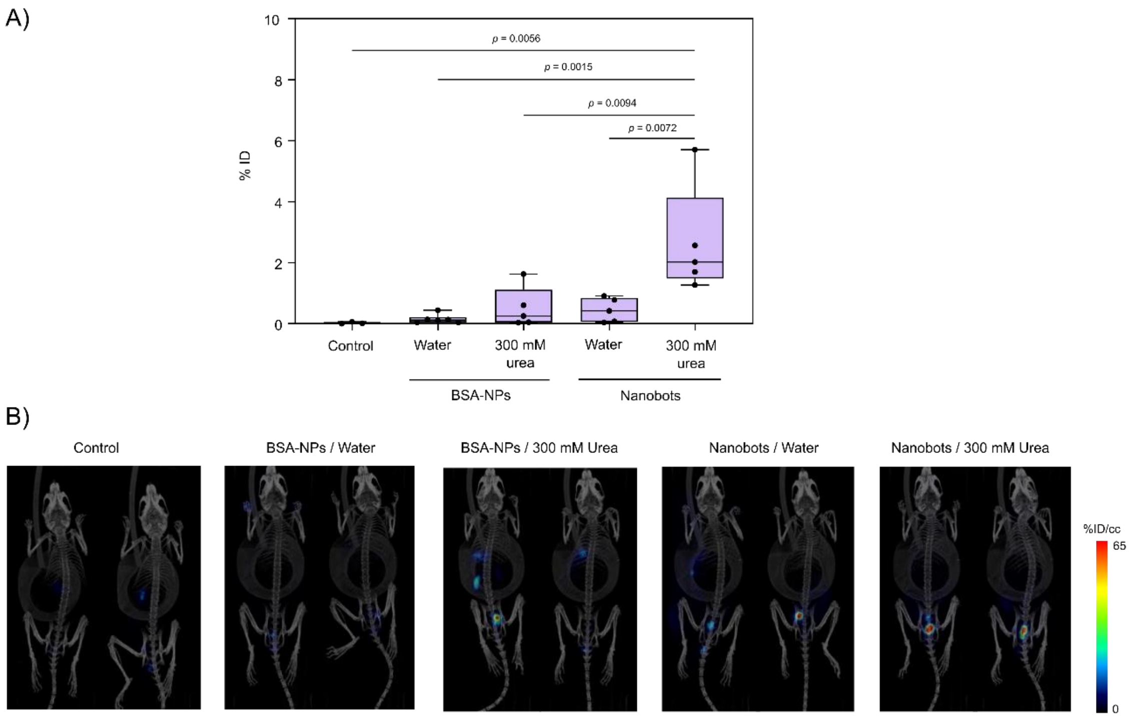

Supplementary Figure S5: Radioactivity accumulation for control (healthy, n=3, biological replicates) animals, and tumor-bearing animals included in all groups (determined by PET, n=5, biological replicates). A) Results are expressed as percentage of injected dose (% ID) as box plots (center line at the median, upper bound at 75th percentile and lower bound at 25th percentile) with whiskers at minimum and maximum values. Each dot represents an individual animal. Statistical analysis performed with one-way ANOVA (p = 0.0056, 0.0015, 0.0094, and 0.0072 respect to Nanobots in urea). B) Representative PET-CT images (maximum intensity projections; coronal view) obtained for representative animals of all groups and control, showing distribution of radioactivity at the whole-body level. Minor presence of radioactivity can be found in some animals in stomach, liver and kidneys, most probably due to animals ingesting residual radioactivity at the administration site during awake period (between administration of the labeled nanobots and imaging time).

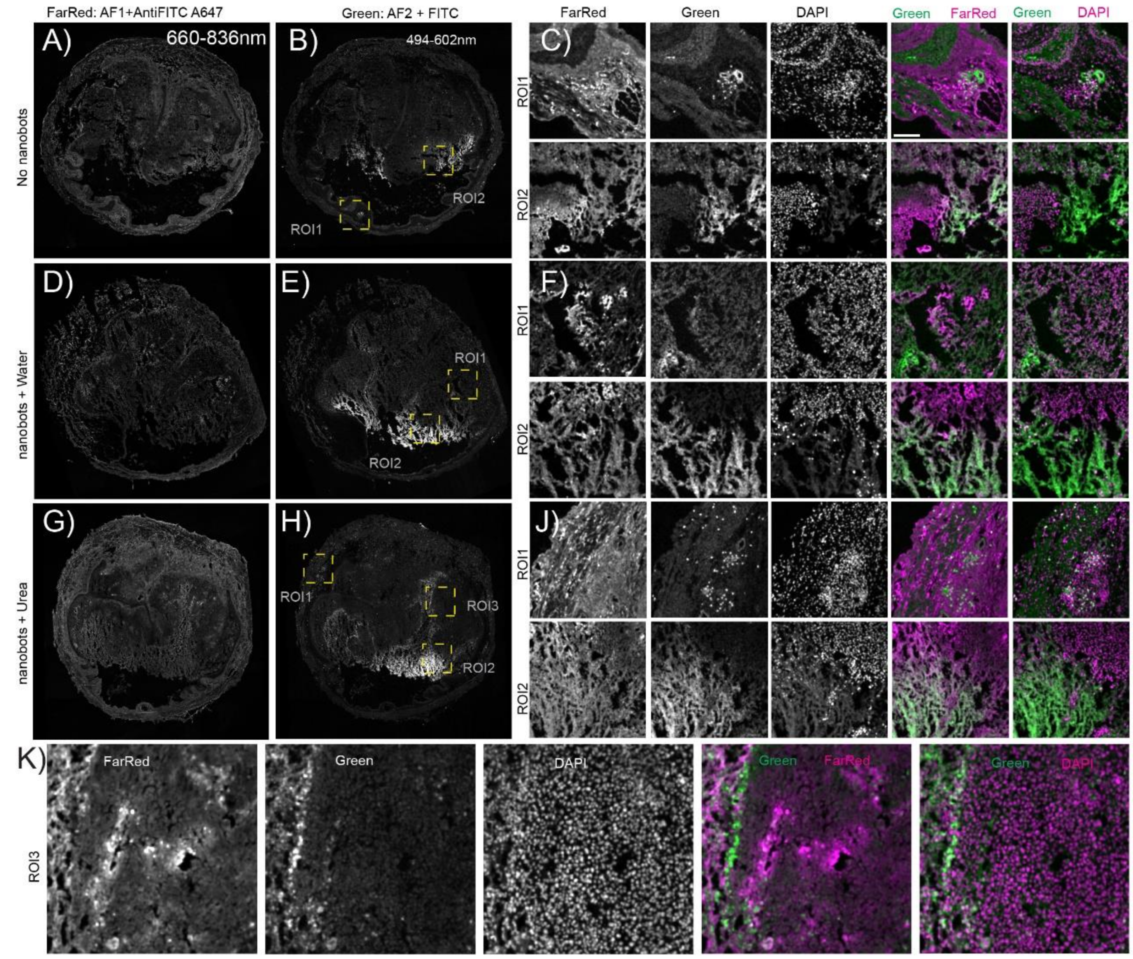

Supplementary Figure S6: Confocal microscopy in *ex vivo* bladders. Representative sections from three distinct animal conditions (middle cut in the organ): A-C: Tumoral bladder without nanobots; D-F: Tumoral bladder with nanobots in water; G-J: Tumoral bladder with nanobots in urea. The following images and insets provide details on nanobot localization and tissue features: A), D), G): Image in the far-red channel (660-836nm) targeting nanobots labeled with anti-FITC Alexa647. B), E), H): Image in the green channel (494-602nm) targeting nanobots labeled with FITC. C), F), I), J): Insets of Regions of Interest (ROI) drawn in B, E, H, with sequences from left to right: Far red, Green, DAPI, Green (in green) with Far red (magenta), and Green (in green) with DAPI (in magenta). ROI2 shows necrotic tissues (with diffuse DAPI labeling) at the intraluminal tumor edge, while ROI1 and ROI3 highlight regions where intensity features in the green channel surpass tissue background. No correlation between the Green and Far-red channels was detected. Scale bar in C: 200 µm. Representative images of experiments repeated with 4 animals for A-B, D-E, 2 animals for G-H (biological replicates). For each animal, 3 distant sections were analyzed.

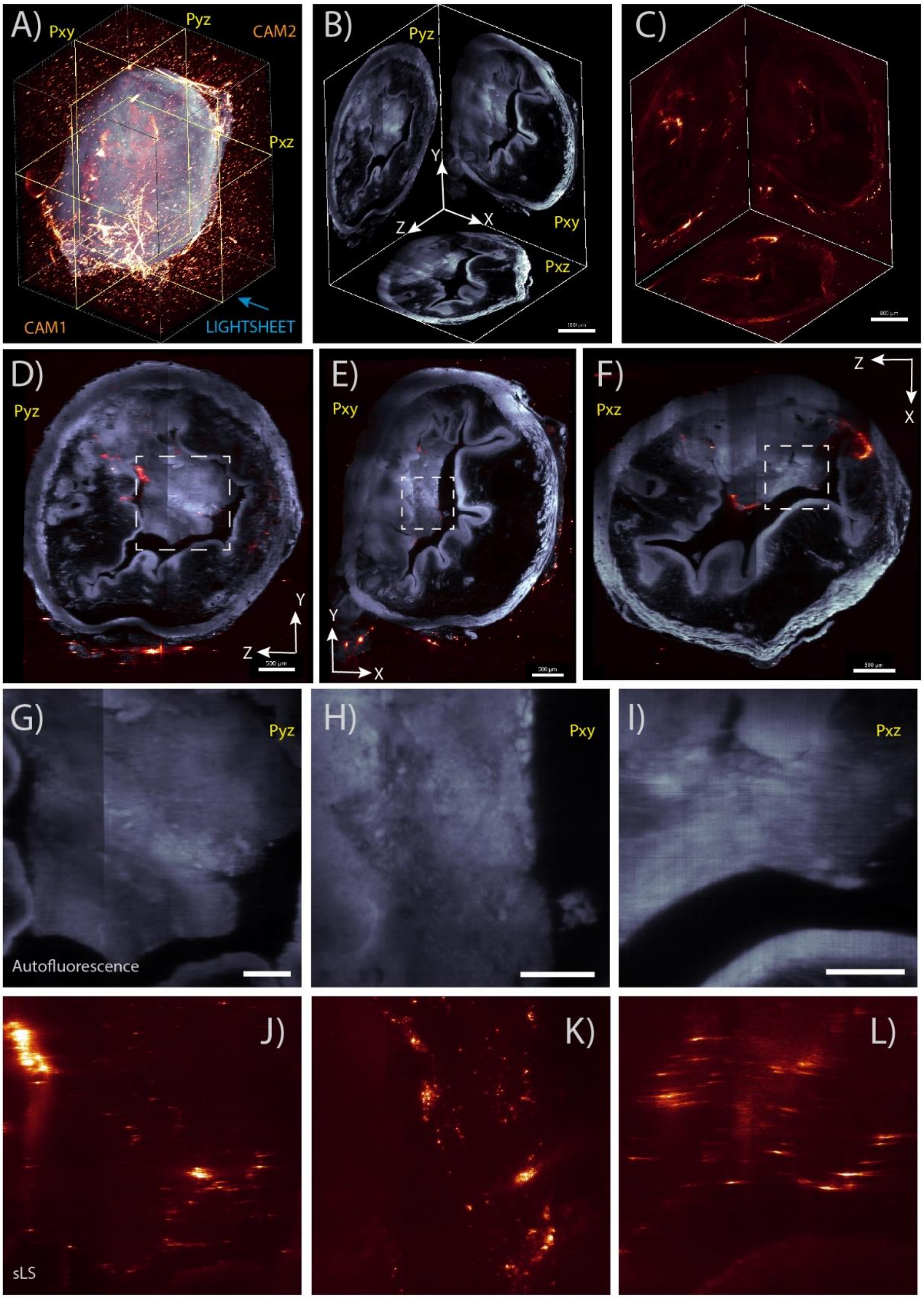

Supplementary Figure S7: Orthogonal views with lightsheet imaging in fluorescence and scattered modes. A) Maximum Intensity Projection of a bladder volume with images in Autofluorescence (Grey) and Scattered sLS (Glow) modes. Orientation of orthogonally sliced planes Pxy, Pxz, Pyz reported in other panels are shown in yellow. CAM1 and CAM2 depict camera positions for detection with opposite cameras along the Z-axis, and the orientation of the lightsheet illumination is indicated by a blue arrow, traveling along the X-axis. B, C) Face-to-face images in planes Pxy, Pxz, Pyz for Autofluorescence (B) and sLS (C). D, E, F) Orthogonal views Pyz, Pxy, and Pxz, respectively. G-L) Cropped views from dashed squares in D-F for Autofluorescence (G-I) and sLS (J-L). Scale bars: A, B,-C: 800 µm; D-F: 500 µm; G-I: 200 µm.

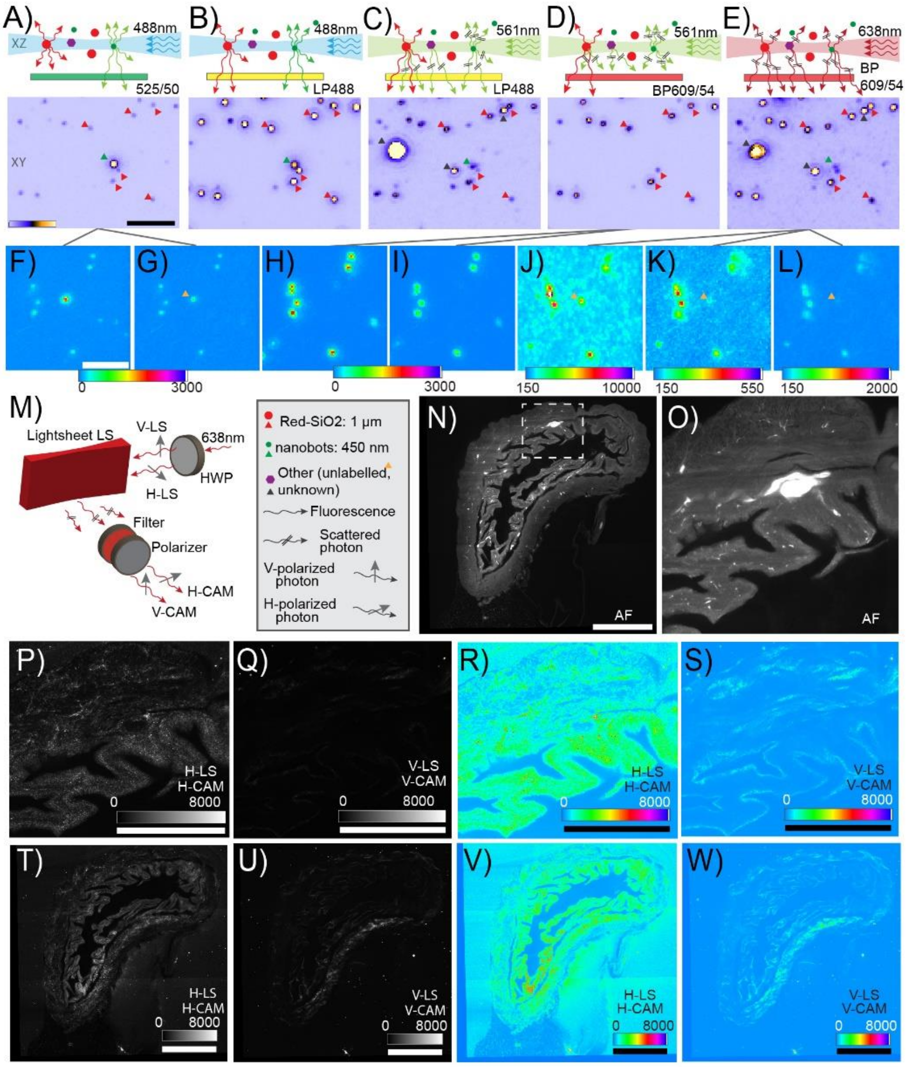

Supplementary Figure S8: Polarized Scattered Lightsheet (sLS) microscopy with nanobots in vitro and in healthy bladder tissues. A-E) FITC-labelled nanobots (450nm) and commercial red silica beads (1 µm) mounted in cleared agarose imaged at 9.6x. Top: lightsheet illumination scheme showing particles (see also M) and laser lines and filters detection; Bottom: maximum intensity projections (MIP) over 125 µm z-depth. nanobots and red beads imaged across three fluorescence channels: green bandpass (A), green/red longpass (B) and red bandpass (D), and with sLS induced at laser 561 nm laser (C) or 638 nm (E). Both particles are detected by sLS, as pointed with green (nanobots) and red (red beads) arrowheads. Dark arrowheads point at unlabeled particles or larger agarose crystals. In A, faint signal originates from crosstalk of red beads into green. F-G) same sample over small 57.5 µm z-depth, strong photobleaching of FITC is shown (orange arrowhead) from F (first time) to G (imaged 6 times). H-I) Red beads before and after 6 repeated stacks. J-L) MIP of sLS signal (same volume) induced by 638 nm, with horizontally polarized illumination (H-LS, in J), vertically polarized (V-LS, in K-L) shown with two intensity scales. M) Polarized sLS: a half-wave plate (HWP) tunes the polarization of the lightsheet (H-LS or V-LS: horizontal means parallel to plane of incidence), and a polarizer is used to select the polarization before the camera (named H-CAM or V-CAM). The box reports symbols for elements in A-E and M. N-O). N-W) Healthy bladder (without tumor, without particles) in autofluorescence (AF): middle plane N, zoomed in O from dashed rectangle. P-W) sLS in the same plane with H-LS and H-CAM, and V-LS and V-CAM. All images shown at the same intensity scale. H-LS induces strong scattering from the whole tissue and agarose, while V-LS induces a weaker response with reduced scattered signal, where only urothelium and some locations in detrusor muscles generate

weak signal. Scale bars: A,F: 20 µm; P-S: 500 µm; N,T-W: 1 mm. Look-up tables (from ImageJ/Fiji): A-F: “ICA”; N-Q, T-U: “Grey”; A-E, F-L, R-S, V-W: “biop12colors”.

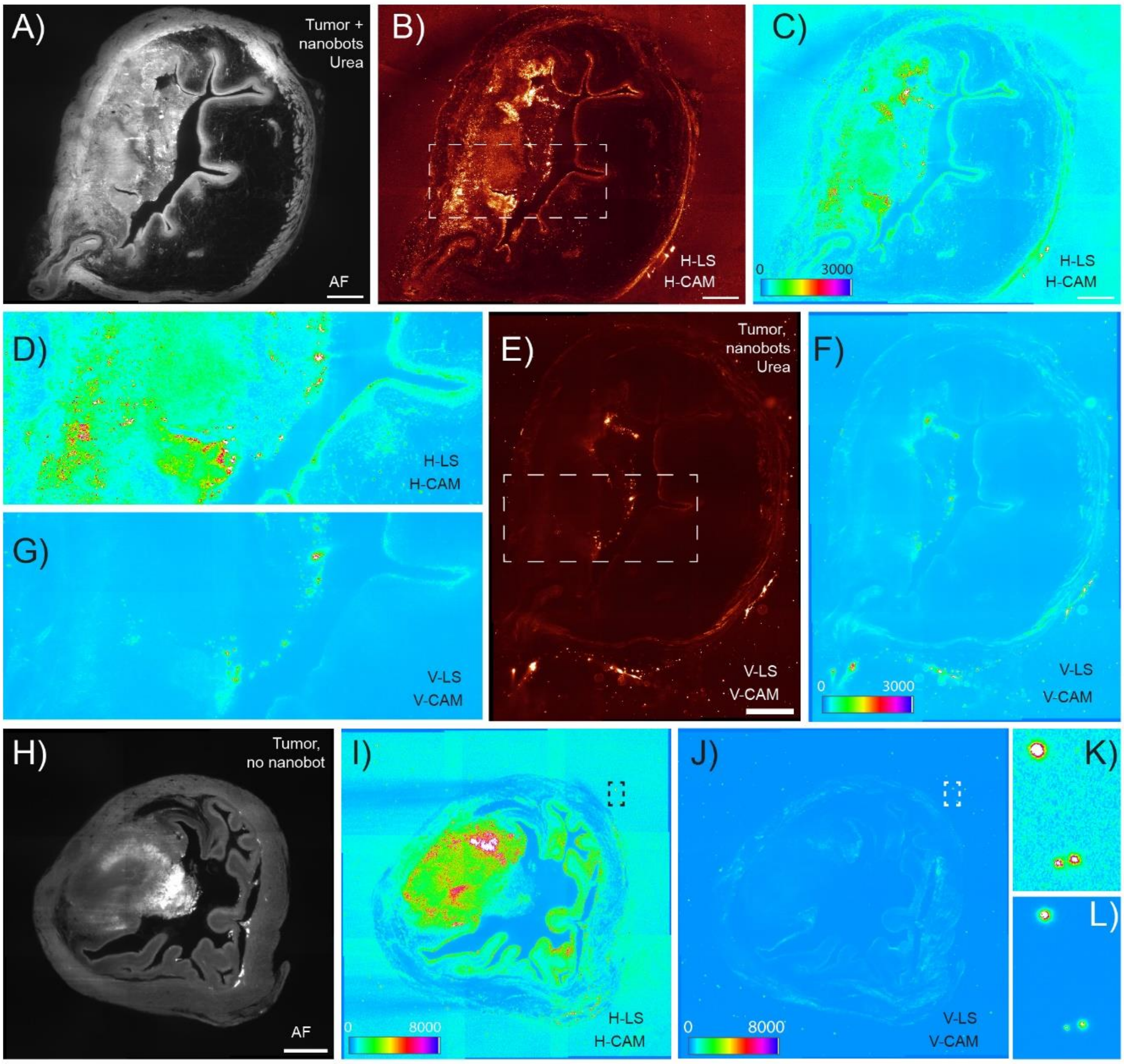

Supplementary Figure S9: Polarized scattered lightsheet (sLS) microscopy reveals nanobots in bladder tissues with tumors. Scattered imaging applied to bladders with tumor, with nanobots (A-F) and without (H-L). A) Autofluorescence in the midplane of the bladder, also shown in Figure 5. B, C) H-LS illumination with H-CAM detection shows strong scattered response in the tumor and in the external agarose block. D) Zoomed from dashed area in B. E, F) V-LS illumination with V-CAM detection shows drastically reduced scattered response in agarose and tumor, but a strongly maintained scattered signal where nanobots are located (as shown in G, zoomed from dashed are in E). H-L) In the control bladder with tumor but without nanobots, H-LS with H-CAM similarly generates strong signal in the tumor and agarose block, but V-LS with V-CAM extinguishes most of this response to leave only residual signal from urothelium and muscles, similar to the healthy tissue control in Fig. S8. K-L: cropped images from I-J dashed area to show the background suppression in the agarose block from H-LS/H-CAM to V-LS/V-CAM, showing the specific suppression of scattered signal when compared to agar crystals, also shown in Fig. S8-C,E that maintain a strong signal. Color scales (ImageJ/Fiji), B, E: “Glow” LUT; C,D,F,G,J-M: “biop12colors” LUT. Scale bars: A,B: 300 µm; E: 600 µm; H: 500 µm.

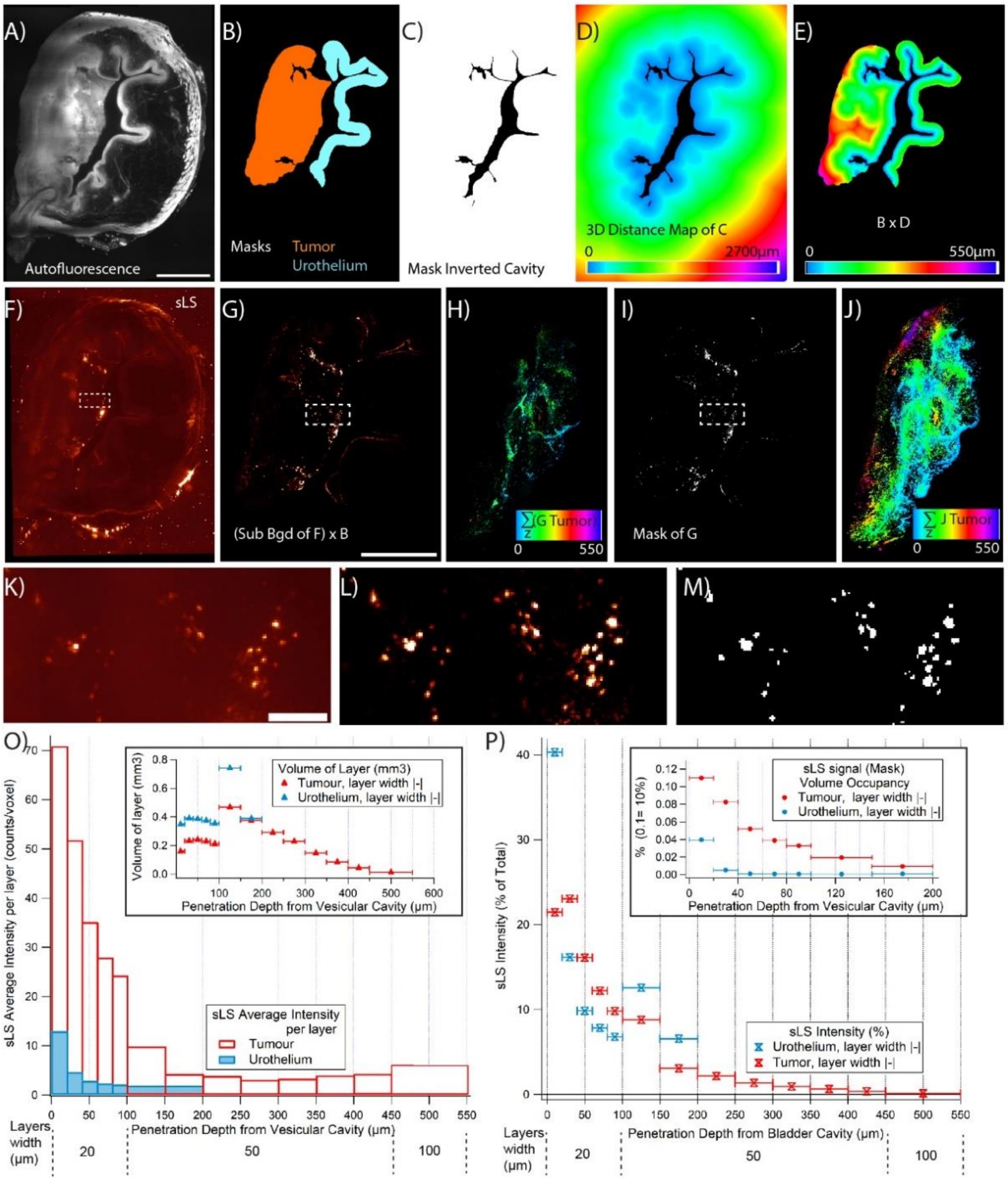

Supplementary Figure S10. Full 3D analysis of sLS signal in Tumor vs Urothelium. A) Single plane in autofluorescence showing tumor (left) and urothelium (right). Scale bar: 1mm. B) Manual 3D annotations of tumor (orange) and urothelium (cyan), and of C) intravesicular lumen/cavity, including possible tissue folds. D) 3D Distance Map computed on the mask in C. E) Isolation of the 3D Distance Map with Tumor and Urothelium masks from B. F) Raw sLS signal in the same plane as in A, and with background subtracted in G. H) Maximum Intensity Projection (MIP) of G (tumor only) with look up table showing quantitatively sLS signal penetration in tumor. I) Mask of sLS signal segmented from G, and J) MIP of the mask with equivalent LUT as in H, expressing visually how deep signal reaches. K, L, M) close-ups from dashed boxes in F, G, I. O) Plots of sLS average intensity in each layer (i.e. integrated density divided by layer volume) and, in inset, layer volume. P) Plots of sLS average intensity percentage to its total detected in all layers (from 0 to 550 µm in 13 layers for tumor, from 0 to 200 µm in 7 layers for Urothelium), and, in inset, sLS volume occupancy, calculated from the volume of the segmented sLS signal over the volume of the layer. Layers have variable width of 20, 50 or 100 µm, see horizontal bars on data points, as shown in the lower scale on both plots. Color scales (ImageJ/Fiji), F, L: “Glow” LUT with black zero; D, E, H, K: “biop12colors” LUT. Scale bars: A) 1 mm, G) 200 µm, K) 20 µm

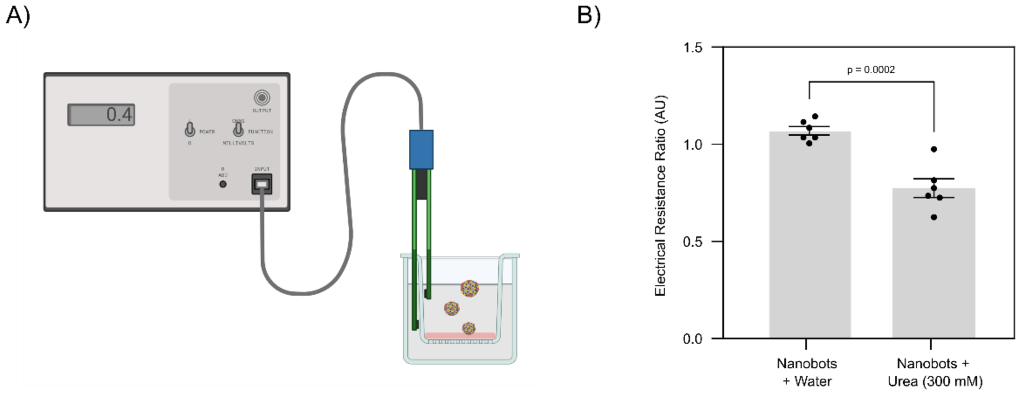

Supplementary Figure S11. Extracellular matrix degradation. A) Schematic representation of the experimental setting using EVOM2 Meter and STX2 chopstick electrodes. Created with BioRender.com B) Electrical resistance ratio of values post-incubation with nanobots in the presence or absence of urea over values pre-incubation (n = 6 independent samples). Data is presented as mean values and error bars represent the standard error of the mean. Statistical analysis performed with a two-sided unpaired t-test (p = 0.0002).

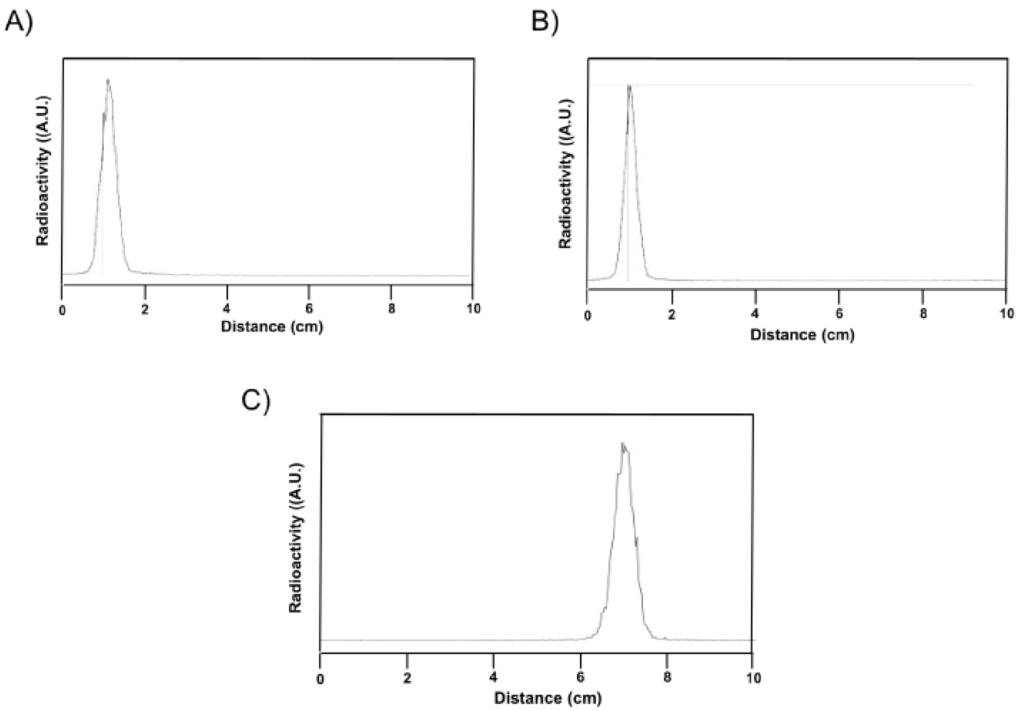

Supplementary Figure S12. Thin layer chromatography profiles obtained after analysis of ^{131}I-nanobots incubated in (A) water and (B) 300 mM aqueous solution of urea for 1 h at 37°C. The presence of a single peak at the seeding point confirms the stability of the labelled nanobots. A control analysis corresponding to free ^{131}I-iodide is shown in (C).

***Supplementary Discussion on* Confocal imaging of nanobots in ex-vivo bladders**

To overcome the resolution of PET imaging, insufficient to obtain in-depth radioactivity profiles in the tumors, we made use of confocal fluorescence microscopy of FITC-urease nanobots to try to determine their distribution within the body of urinary bladder and penetration to the tumor. Additionally, hypothesizing that FITC would conflict with tissue autofluorescence, nanobots were further labelled with a secondary far red Alexa647 fluorophore. 10 female mice were inoculated intravesically with MB49 cells, and tumor growth was monitored at days 7 and 14 after cell implantation by MRI, following the same procedure described above. For confocal fluorescence microscopy, animals were randomized in three groups: (1) FITC-urease nanobots administered in ultrapure water; (2) FITC-urease nanobots administered in 300 mM urea solution in water; and (3) absence of nanobots (control). The procedure of administration of FITC-nanobots was the same described for PET imaging studies (1 h incubation). After 3 h post-administration, animals were sacrificed, and the bladders were collected, frozen and analyzed by confocal fluorescence microscopy.

Representative confocal images of thin (10 μm) histological slices (middle plane of the organ) of the three cases are displayed in Supp. Fig. S6. Nanobots were stained with a secondary anti-FITC Alexa Fluor 647 to circumvent the high tissue autofluorescence, which interferes with the direct staining of FITC positive nanobots, as shown in Supp. Fig. S6 A,D,G. All histological sections were labelled identically, and three sections per animal (caudal, middle and cranial cuts) were imaged for all animals treated (4 animals for nanobots with urea, 4 with nanobots in water, 2 controls without nanobots). Comparison of image channels targeted to FITC and Alexa647 unfortunately did not reveal any signal correlation in the three cases (shown in Supp. Fig. S6, color panels in C, F, I, J: with urea, with water, without nanobots). In particular, by qualitatively analyzing the highest intensity areas in images (i.e. above background, represented in Supp. Fig. S6 insets C, F, I, J), we observed, on one hand, that high

levels of autofluorescence are induced in important portions of tumor tissues, in all conditions, where DAPI labelling seemed to fail, hence suggesting the presence of necrotic tissue that may shield the sensitivity of the imaging in the green channel, precisely where FITC-nanobots were hoped to be detected. On the other hand, no significant correlation between green (for FITC) and far red (for anti-FITC) channels could be visualized, suggesting that none of the high intensities could be attributed to nanobots, in all sections, In particular, the comparison of control animals without nanobots to other animals with nanobots yielded similar observations, hence confirming that fluorescence imaging did not enable to detect nanobots specifically with sufficient confidence. From this outcome, we concluded that immunolabelling targeting FITC in nanoparticles is not reliable, and we sought an alternative label free optical technique to detect nanobots, on one hand to circumvent the low fluorescence levels issues, and on the other hand to improve on the potential issue of sparse nanobots localization in 2D histological sections, for which those preliminary results did not encourage us to pursue the costly pipeline of labelling and imaging all sections of bladders.

Supplementary discussion **on Polarized Scattered Light sheet (sLS) microscopy to detect nanobots in ex-vivo bladder tissues**

To detect 450nm-sized nanobots by sLS in whole tumoral bladders, we first optimized sLS to reveal nanobots specifically against background noise of reflected laser light. We implemented sLS imaging with polarization control both in illumination *and* in detection, following previous work[1] that suggested this approach as a way to enhance image quality and signal-to-noise-ratio (SNR) for label-free sLS imaging. Herein and in Supplementary Figures S8-S9, we refer to lightsheet illumination with horizontal (H-LS) and vertical (V-LS) polarizations, respectively, and to H-CAM and V-CAM for a horizontally and vertically

[1] Di Battista D, Merino D, Zacharakis G, et al. Enhanced Light Sheet Elastic Scattering Microscopy by Using a Supercontinuum Laser. (2019) Methods Protoc *2*(3), 57

polarized detection (CAM: Camera). For convenience, we report the use of two configurations that we name H-H for horizontally polarized illumination and detection, and V-V for vertical polarized illumination and detection, as shown with the optical configuration in Fig. Supp S8 M). Here, we aimed to find the best polarization combination that could enhance nanobots detection in vitro in cleared agarose blocks, and we demonstrate that "vertically-oriented" polarization, both in illumination and detection (V-LS and V-CAM, respectively, or V-V, in Supp. Fig S8M), yields imaging in tissues with the lowest laser background noise conditions.

We started with comparing sLS detection efficiency with FITC-labelled nanobots (450nm in size) and commercial red-fluorescent silica particles (1 µm) mounted in agarose blocks, cleared prior to imaging. We could show (Supp. Fig. S8A-E) that even at moderate magnification (9.6x), both types of particles were detectable in fluorescence (Supp Fig S8A, B, D, F-I) and sLS (Supp Fig S8 C, E, J-L), although FITC nanobots featured a very high instability with fast photobleaching after few frames in a short time lapse (6 time points: Supp. Fig. S8 F-G). We also showed that V-LS illumination enables to decrease scattered signal from the agarose, and to increase the specific Signal-to-Noise-Ratio (SNR) of nanobots (Supp. Fig. S8 J-L).

Next we characterized cleared bladder tissues under sLS, with and without tumor, and with and without nanobots. In a healthy bladder tissue (Supp. Fig. S8 N-W), we observed that sLS imaging provides a label-free image, complementary to autofluorescence imaging, and that the scattered signal is much stronger with H-H than with V-V. However, a careful comparison of the two conditions showed that the bladder tissues, here *without* nanoparticles, answer in a complex way to changes in polarization: the background sLS signal does not simply decrease uniformly with the change of illumination polarization, but rather follows a tissue-specific response. More specifically, we show that H-H induces the collection of strong sLS signal both in the tissue and the surrounding agarose (Supp. Fig. S8 R, V), while the V-V configuration

attenuates most of this signal, but leaves residual sLS signal emanating from the urothelium and muscle tissues (Supp. Fig. S8 S-W).

The explanation of this phenomenon is beyond the scope of this study, but we conclude here that polarization control in sLS may enable, on one hand, to enhance the response of nanoparticles by decreasing nonspecific background scattered light (as shown in agarose (Supp. Fig. S8 K-M), and on the other hand that a similar signal enhancement could be obtained in cleared tissues, keeping in mind that other structures may also display a polarization-specific response.

Next, we addressed whether this effect can be exploited to improve the detection of nanobots in bladder tissues with and without tumors. The H-H to V-V sLS signal comparison in a bladder with tumor showed (Supp. Fig. S9, H-L) that H-H signal emanating specifically from a tumor tissue is actually even stronger than in the surrounding healthy tissue. However, using V-V configuration we could show that tumor-specific signal can be totally damped (Supp. Fig. S9 J), while residual sLS signal from urothelium and detrusor muscles are still visible, similar to the case of healthy bladder (previously in Supp Fig S8). When repeating the experiment in a bladder with tumor and injected urease nanobots, we now observed that, again, the strong sLS response of the tumor visible in H-H is greatly attenuated with V-V (in Supp. Fig. S9, compare D and G), however with the striking difference that specific signal from nanobots remains observable. This is shown in Supp Fig. S9 B-F, in a single optical section, where enriched signal emanates from the tumor surface, while scattered signal heavily drops from H-H to V-V in the tumor body, the Lamina propria and the surrounding agarose. Noteworthy, as shown previously with nanobots in Agarose blocks (Supp. Fig S8), foci of scattered signal in Agarose do remain visible with V-V while the background in the agarose damps (Supp. Fig. S9 K,L), suggesting that the polarization dependence of the scattered signal could be conditioned by the characteristic scale and local structure of the scatterers.

To confirm that V-V sLS imaging enables to enhance nanobots scattered signal, we then compared the total 3D intensity of scattered signal in bladder tissues with tumor and Urease-nanobots in urea- (Fig. 4 A,B), with tumor and urease nanobots in water (Fig. 4 C,D), with tumor and without nanobots (Fig. 4 E,F), in a healthy bladder without tumor and particles (Fig. 4 I,J), and in healthy bladder with nanobots in urea (Fig. 4 G,H). Importantly, we analyzed the scattered signal in a masked volume (see Methods) that includes bladder inner tissues (urothelium, Lamina propria, tumor when present) but that excludes the inner cavity *and* the Detrusor muscles, as the latter were shown to induce specific scattered signal (in Supp Fig. S8). Intensity profiles in Fig. 4 K,L clearly show that the presence of nanobots in urea induces very high levels of measured signal, both in a bladder with or without tumor, while tissue without particles show consistently similar distributions of very low intensities. Comparing nanobots in urea in bladder with tumor and without, we clearly observed foci of high intensities in both, but a vastly more enriched distribution around the tumor. Interestingly, in the bladder with tumor and nanobots in water (Fig. 4C,D), the intensity distribution was similar to that of tissues without particles, with no observable foci standing out from the background signal of the tissue.

In summary, we showed that a suitable polarization configuration enables to almost completely extinguish the dominant laser scattered background from both tissue and surrounding agarose, in healthy bladders (Supp. Fig. S8 N-W) and in particular in tumoral bladder (Supp. Fig. S9 H-L), whereas unsuitable, uncontrolled or random polarization configurations may generate a very high scattered light response in the tumor, hence summing to the particles signal and making them undetectable specifically. In turn, we could show that in bladders *with* nanobots, an enhanced nanobot-specific scattered signal can be revealed while most of the background signal of tissue or tumor can be damped (Supp. Fig. S9). From this consistent outcome, we conclude that the V-V polarization configuration in scattered lightsheet

microscopy enables to detect nanobots in whole bladders by enhancing their response into clearly visible foci of scattered light that stands out from tissue background signal.

***Supplementary analysis* of sLS signal distribution in whole tumor vs. urothelium**

To complement the analysis reported in Figure 3, where the sLS signal was estimated only along three concentric layers from the surface of the tumor, where the tumor boundaries are unambiguously detectable, we have here performed a similar analysis in the whole volume of the tumor (Suppl. Fig. S10). To achieve this, we manually and more subjectively annotated the tumor volume, based on the autofluorescence signal, by aiming to detect all lesions in the bladder, and taking as a reference the visual aspect of the healthy urothelium and lamina propia within the same organ. The primary aim of this annotation process was to estimate the precise location of the tumor surface exposed to the vesicular lumen, which is an inherently more difficult task because the fixed organ is imaged in an empty and folded state compared to its *in vivo* state that is filled with liquid, hence limiting our ability to precisely detect every single tissue fold or intravesicular space. Nevertheless, assuming this process is performed rigorously within the experimental limitations of the task (resolution, autofluorescence specificity), we performed full 3D quantification of the sLS signal in the tumor and the urothelium. We first estimated the depth inside the tumour as the shortest distance to the intravesicular cavity, calculated with a 3D Distance Map tool (see methods). Once this value computed in 3D, we used it to dissect the full volume in 7 and 13 layers (of variable thickness) by image segmentation for urothelium and tumor respectively, as shown in Suppl. Fig. S10 A-E where the full layer depth estimation workflow is illustrated. We then quantified the sLS intensity in each layer (Fig. S10 F-H, K, L), as well as the segmented volume of sLS which shows the volume occupied by nanoparticles (Fig. S10 I, J, M). The results are plotted in Fig. S10 O-P, from which we interpret further interesting 3D insights about nanobots distribution in tumor: firstly, we can observe that nanobots prominently accumulate at the tumor compared to the

urothelium with a 5.5-fold richer nanobots accumulation in the first layer (0-20 µm, Fig. S10-O), reaching up to 12-fold difference in the following layers (20-40, 40-60, 60-80, 80-100 µm), and an average of 6.9-fold difference across the layers 0-200 µm. Calculating the median of penetration, expressing the depth at which half of the signal is detected, also shows a strong difference with 35.6 µm in the tumor against 22.2 µm in the urothelium, suggesting that nanobots penetrate deeper into tumoral tissues. This is also observed by quantifying the amount of signal to the total across layers 200-550 µm, that still gathers more than 5.5% of the total detected sLS signal. Secondly, we also computed the volume occupied by the sLS signal, obtained by 3D segmentation, compared to the layer volume, hence yielding a volume occupancy that expresses how much nanobots fill the volume of the tissues. In Fig. S10 P, we report that nanobots fill up to 11% of the first layer 0-20 µm against 3.9% in the urothelium, and more strikingly 5.2% against 0.1% in the deeper layer 40-60 µm. Overall, in layers 0-200 µm, the average occupancy of sLS reached 4.9% against 0.7% in the urothelium.

Supplementary Methods

Tracer particle motion dynamics through optical microscopy

Optical microscopy videos were acquired at 25 frames per second using a Leica DMi8 microscope, coupled with a Hamamatsu high-speed CCD camera and a 10X objective. To do so, 2-micron-sized polystyrene particles, at a final concentration of 0.001% (w/v), were evenly dispersed in PBS or 300 mM urea solution. Afterward, a Petri dish was filled with 3 ml and the motion of the tracer particles was recorded in the microscope. A 5 µl drop with nanobots (20 mg/ml) was then added to the liquid-filled Petri dish and videos were recorded, showing the swarm on the top-right corner of the field of view while simultaneously observing the motion of the tracer particles. At least 20 tracer particles were analyzed per condition using a Python-based code to obtain their trajectories and calculate the mean-squared displacement.

In toto tumor analysis of nanobots localization

The same suite of macros described in Methods was used to analyze the 3D localization of sLS nanobots signal in 3D in the full tumor, shown in Fig. S10, although here the vesicular cavity/lumen was annotated more precisely in 3D by increasing the number of annotations (every 9 planes, 22.5µm spacing), intending to reveal every possible fold or lumen space in the bladder, where detectable. The resulting mask was used to compute a 3D distance map of the shortest distances to the cavity using ImageJ/3DSuite[2]. Similarly, the full tumor extent was annotated by selecting regions with higher/denser textures in autofluorescence compared to the urothelium/lamina propria. The urothelium was also manually annotated. Final 3D tumor and urothelium distance maps were obtained by combining the resulting masks. Segmentation of the sLS signal was performed after background subtraction (radius 5) and fixed thresholding.

[2] Ollion J, Cochennec J, Loll F, Escudé C, Boudier T. TANGO: a generic tool for high-throughput 3D image analysis for studying nuclear organization. Bioinformatics 2013, 29: 1840 - 1841.

Quantities reported in Fig. S10 are based on measuring integrated densities (summed intensities) of the masks (to estimate volume) and sLS signal using subsampled images with isotropic voxel sizes of 3.385µm. Volume occupancy is defined by the ratio between segmented sLS mask volume and tumor or urothelium mask/layer volumes.

Electrical resistance measurements

60 µl of Corning® Matrigel® Matrix were added into 24-well ThinCert™ cell culture inserts (Greiner Bio-One®) and left for 30 min at 37 ºC. 200 µl and 600 µl of MilliQ H_2O were added to the upper and lower compartment, respectively, and left for 10 min at room temperature. Electrical resistance was measured using an EVOM2 voltohmmeter with a chopstick STX2 electrode (World Precision Instruments). Then, nanobots were added at a final concentration of 30 µg/ml with or without 300 mM of urea. After an incubation of 1 h at 37 ºC, electrical resistance was measured again and the ratio of post- over pre-incubation values was calculated.
