# Supplementary material for: Urease-powered nanobots for radionuclide bladder cancer therapy
Source: Nat Nanotechnol. 2024 Jan 15;19(4):554–64. doi: 10.1038/s41565-023-01577-y (PMC11026160; doi:10.1038/s41565-023-01577-y)
Supplement: Supplementary file 2 — Reporting Summary [file 41565_2023_1577_MOESM2_ESM.pdf]

nature portfolio

Corresponding author(s): Samuel Sánchez, Jordi Llop, Julien Colombelli

Last updated by author(s): Nov 14, 2023

Reporting Summary

Nature Portfolio wishes to improve the reproducibility of the work that we publish. This form provides structure for consistency and transparency in reporting. For further information on Nature Portfolio policies, see our Editorial Policies and the Editorial Policy Checklist.

Statistics

For all statistical analyses, confirm that the following items are present in the figure legend, table legend, main text, or Methods section.

n/a	Confirmed	
☐	☒	The exact sample size (*n*) for each experimental group/condition, given as a discrete number and unit of measurement
☐	☒	A statement on whether measurements were taken from distinct samples or whether the same sample was measured repeatedly
☐	☒	The statistical test(s) used AND whether they are one- or two-sided<br>*Only common tests should be described solely by name; describe more complex techniques in the Methods section.*
☐	☒	A description of all covariates tested
☐	☒	A description of any assumptions or corrections, such as tests of normality and adjustment for multiple comparisons
☐	☒	A full description of the statistical parameters including central tendency (e.g. means) or other basic estimates (e.g. regression coefficient) AND variation (e.g. standard deviation) or associated estimates of uncertainty (e.g. confidence intervals)
☐	☒	For null hypothesis testing, the test statistic (e.g. *F*, *t*, *r*) with confidence intervals, effect sizes, degrees of freedom and *P* value noted<br>*Give P values as exact values whenever suitable.*
☒	☐	For Bayesian analysis, information on the choice of priors and Markov chain Monte Carlo settings
☒	☐	For hierarchical and complex designs, identification of the appropriate level for tests and full reporting of outcomes
☒	☐	Estimates of effect sizes (e.g. Cohen's *d*, Pearson's *r*), indicating how they were calculated

Our web collection on statistics for biologists contains articles on many of the points above.

Software and code

Policy information about availability of computer code

Data collection	Quantification of Lightsheet images was performed using custom ImageJ macro scripts that facilitate user-guided segmentation, particularly for datasets exceeding available RAM (typically >500GB). These macros, developed in the ImageJ Macro language, were executed using Fiji with IJ version 2.3.0/1.53d. As mentioned in the manuscript, the full set of macros is part of a comprehensive suite for 'Large Image Analysis' and will be detailed in a separate technical article. Consequently, the code will be made available upon reasonable request.
Data analysis	PMOD image processing tool (version 4.3) for PET image analysis. Statistical analyses were performed with GraphPad Prism (version 8). Videos were generated with Imaris (pay version) 9.1.0. Figure panels were generated with Imaris Viewer (free version) 9.9.1

For manuscripts utilizing custom algorithms or software that are central to the research but not yet described in published literature, software must be made available to editors and reviewers. We strongly encourage code deposition in a community repository (e.g. GitHub). See the Nature Portfolio guidelines for submitting code & software for further information.

Data

Policy information about availability of data

All manuscripts must include a data availability statement. This statement should provide the following information, where applicable:

- Accession codes, unique identifiers, or web links for publicly available datasets
- A description of any restrictions on data availability
- For clinical datasets or third party data, please ensure that the statement adheres to our policy

The data supporting the findings of this study are available within the paper as Source Data. The unprocessed raw image data from PET, CT, sLS and MRI are available from the authors upon request.

Human research participants

Policy information about studies involving human research participants and Sex and Gender in Research.

Reporting on sex and gender	Not applied
Population characteristics	Not applied
Recruitment	Not applied
Ethics oversight	Not applied

Note that full information on the approval of the study protocol must also be provided in the manuscript.

Field-specific reporting

Please select the one below that is the best fit for your research. If you are not sure, read the appropriate sections before making your selection.

☒ Life sciences ☐ Behavioural & social sciences ☐ Ecological, evolutionary & environmental sciences

For a reference copy of the document with all sections, see nature.com/documents/nr-reporting-summary-flat.pdf

Life sciences study design

All studies must disclose on these points even when the disclosure is negative.

Sample size	Sample was decided according to the resource equation, and considering the previous results obtained by the group in similar experiments.
Data exclusions	All animals were initially included into the experiment. Besides, criteria of excluding animals were established a priori. Animals were excluded when the tumour was not established on the bladder, or mice die due to anaesthesia.
Replication	All experiments included repetitions and/or technical replicates to verify result reproducibility.
Randomization	In vivo experiments involved the randomization of samples. Initially, mice were randomly assigned to cages to achieve the appropriate 'n' for each experimental group based on their weight. Following tumor induction, mice were further distributed based on the size of the tumor analyzed by MRI.
Blinding	In animal experiments aimed at determining tumor accumulation of nanobots using PET, researchers analyzing the images were blinded to the administered nanobots. Similarly, in animal experiments to assess therapeutic efficacy, researchers determining tumor volume using MRI were blinded to the treatment.

Reporting for specific materials, systems and methods

We require information from authors about some types of materials, experimental systems and methods used in many studies. Here, indicate whether each material, system or method listed is relevant to your study. If you are not sure if a list item applies to your research, read the appropriate section before selecting a response.

Materials & experimental systems

n/a	Involved in the study	
☐	☒	Antibodies
☐	☒	Eukaryotic cell lines
☒	☐	Palaeontology and archaeology
☐	☒	Animals and other organisms
☒	☐	Clinical data
☒	☐	Dual use research of concern

Methods

n/a	Involved in the study	
☒	☐	ChIP-seq
☒	☐	Flow cytometry
☐	☒	MRI-based neuroimaging

Antibodies

Antibodies used	Mouse Anti-FITC antibody [F4/1] (ab112511, Abcam) and Donkey anti-Mouse IgG (H+L) Highly Cross-Adsorbed Secondary Antibody, Alexa Fluor™ Plus 647 (A32787, Thermo Fisher Scientific)
Validation	Poeter M et al. Annexin A8 controls leukocyte recruitment to activated endothelial cells via cell surface delivery of CD63. Nat Commun 5:3738 (2014). Specialized astrocytes mediate glutamatergic gliotransmission in the CNS. Nature (2023)<br>Prosaposin maintains lipid homeostasis in dopamine neurons and counteracts experimental parkinsonism in rodents. Nat Commun (2023)<br>Good Z et al. Proliferation tracing with single-cell mass cytometry optimizes generation of stem cell memory-like T cells. Nat Biotechnol 37:259-266 (2019).

Eukaryotic cell lines

Policy information about cell lines and Sex and Gender in Research

Cell line source(s)	MB49 tumor bladder cell line was obtained from Dr. Mangsbo and Dr. Tötterman from Rudbeck Laboratory (Uppsala University, Sweden). The MB49 cell line is available through Merck with the reference SCC148.
Authentication	The cell line were not authenticated.
Mycoplasma contamination	The MB49 cell line was tested and found to be negative for the presence of Mycoplasma contamination.
Commonly misidentified lines (See ICLAC register)	No commonly misidentified cell lines were used in the study.

Animals and other research organisms

Policy information about studies involving animals; ARRIVE guidelines recommended for reporting animal research, and Sex and Gender in Research

Laboratory animals	C57BL/6JRj female mice of 8 weeks old purchased from Janvier labs (Le Genest-Saint-Isle, France). The animals were housed in polycarbonate cages with bedding (5 animals per cage). The housing rooms were maintained within the appropriate temperature and relative humidity ranges for rodents (20-24°C and 40-70%, respectively). The lighting in the rooms consisted of 12/12-hour light/dark cycles. The animals were fed with maintenance diet and provided with ad libitum access to food and drinking water.
Wild animals	No wild animals were used in the study.
Reporting on sex	The mouse model used in the present experiments is suitable to study bladder cancer because is orthotopic and syngeneic. However, only female mice were used because male anatomic characteristics make mycobacterial treatment impractical and, differences in sex could influence the response to treatment.
Field-collected samples	No field collected samples were used in the study.
Ethics oversight	The experimental plan was approved by the Ethical Committee at CIC biomaGUNE (project code: AE-biomaGUNE-0820), an external Ethical Committee (Organo Habilitado del IIS Biodonostia; project code: OH20 -018) and by local authorities (Diputación Foral de Guipuzcoa; project code: PRO-AE-SS-184).

Note that full information on the approval of the study protocol must also be provided in the manuscript.

Magnetic resonance imaging

Experimental design

Design type	Anatomical Imaging (Diffusion Weighted Imaging)
Design specifications	Two imaging sessions per animal (7 and 14 days after tumoral cells implantation).

Behavioral performance measures: No behavioral performances were measured in this study

Acquisition

Imaging type(s): Anatomical Imaging (Diffusion Weighted Imaging)

Field strength: 7 T

Sequence & imaging parameters: Spin-echo Difussion Weighted Imaging pulse sequence (SE-DWI) with the following paramenters: TE = 22.3 ms, TR = 2500 ms, n = 2 averages, 1 A0 image (b=0 s mm-2) and one DW image acquired using diffusion gradients in (1, 0, 0) direction with δ = 4.5 ms and Δ = 10.6 ms, giving b = 650 s mm-2 , a 16×16 mm2 field of view, image matrix size of 160×160points, 20 consecutive slices of 0.5 mm thickness (no gap, acquired in interleaved mode), and a bandwidth 493 of 192.9 Hz/pixel.

Area of acquisition: Abdomen at the region of the bladder

Diffusion MRI ☒ Used ☐ Not used

Parameters: Single direction (1,0,0) difusional gradients witg b=0 and b=650 s mm-2. No gating required

Preprocessing

Preprocessing software: Acquisition with Bruker's ParaVIsion 7.0

Normalization: N/A

Normalization template: N/A

Noise and artifact removal: Denoising with DIPY algorithm for Python

Volume censoring: N/A

Statistical modeling & inference

Model type and settings: N/A

Effect(s) tested: N/A

Specify type of analysis: ☐ Whole brain ☒ ROI-based ☐ Both

Anatomical location(s): Bladder

Statistic type for inference (See Eklund et al. 2016): N/A

Correction: N/A

Models & analysis

n/a	Involved in the study
☒	☐ Functional and/or effective connectivity
☒	☐ Graph analysis
☒	☐ Multivariate modeling or predictive analysis
